# Genome-Wide Fitness Test and Mechanism-of-Action Studies of Inhibitory Compounds in Candida albicans

**DOI:** 10.1371/journal.ppat.0030092

**Published:** 2007-06-29

**Authors:** Deming Xu, Bo Jiang, Troy Ketela, Sebastien Lemieux, Karynn Veillette, Nick Martel, John Davison, Susan Sillaots, Steve Trosok, Catherine Bachewich, Howard Bussey, Phil Youngman, Terry Roemer

**Affiliations:** 1 Center of Fungal Genetics, Merck Frosst Canada Ltd., Montreal, Quebec, Canada; 2 Infinity Pharmaceuticals, Cambridge, Massachusetts, United States of America; 3 Institute of Research in Immunology and Cancer, University of Montreal, Montreal, Quebec, Canada; 4 Department of Biology, Concordia University, Montreal, Quebec, Canada; 5 Department of Biology, McGill University, Montreal, Quebec, Canada; 6 Department of Infectious Disease, Merck & Co., Inc., Rahway, New Jersey, United States of America; Johns Hopkins University, United States of America

## Abstract

Candida albicans is a prevalent fungal pathogen amongst the immunocompromised population, causing both superficial and life-threatening infections. Since C. albicans is diploid, classical transmission genetics can not be performed to study specific aspects of its biology and pathogenesis. Here, we exploit the diploid status of C. albicans by constructing a library of 2,868 heterozygous deletion mutants and screening this collection using 35 known or novel compounds to survey chemically induced haploinsufficiency in the pathogen. In this reverse genetic assay termed the fitness test, genes related to the mechanism of action of the probe compounds are clearly identified, supporting their functional roles and genetic interactions. In this report, chemical–genetic relationships are provided for multiple FDA-approved antifungal drugs (fluconazole, voriconazole, caspofungin, 5-fluorocytosine, and amphotericin B) as well as additional compounds targeting ergosterol, fatty acid and sphingolipid biosynthesis, microtubules, actin, secretion, rRNA processing, translation, glycosylation, and protein folding mechanisms. We also demonstrate how chemically induced haploinsufficiency profiles can be used to identify the mechanism of action of novel antifungal agents, thereby illustrating the potential utility of this approach to antifungal drug discovery.

## Author Summary

Candida albicans is the principal human fungal pathogen responsible for life-threatening fungal infections. Despite an urgent need for more efficacious antifungal agents, the pace of discovery has waned using the traditional approaches. In part, this reflects the longstanding limitation of performing mechanism-of-action–based screening directly in those key fungal pathogens for which new antifungal agents are sought. Here we describe an alternative approach, first developed in Saccharomyces cerevisiae and termed the fitness test, to survey approximately 45% of the C. albicans genome for the molecular targets of growth inhibitory compounds. We demonstrate that mechanistically characterized compounds can be used as chemical probes to assist gene function annotations in C. albicans. Similarly, fitness tests performed using newly discovered compounds provide powerful insights into their mechanism of action and therapeutic potential as antifungal agents. Extending this screening paradigm to C. albicans facilitates a pathogen-focused approach to antifungal drug discovery.

## Introduction

Candida albicans is responsible for approximately 50% of all human life-threatening nosocomial fungal infections [1,2]. Completion of its diploid genome sequence [3,4] now provides a foundation for studies on C. albicans biology and pathogenesis, and offers new opportunities for therapeutic intervention. Critical to such activities, however, remains the task of functionally annotating the C. albicans genome. To date, genomic studies reveal significant differences in genomic organization [3–5] and gene essentiality [6] between C. albicans and Saccharomyces cerevisiae. In part, these differences reflect their evolutionary divergence and distinct lifestyle as an opportunistic fungal pathogen versus a saprophytic yeast, respectively. Unlike *S. cerevisiae,* a major impediment to large-scale genetic analyses in C. albicans is the limited ability to perform classical genetic screens, due to its natural diploid state and lack of an easily manipulated sexual cycle. Thus, alternative genetic strategies are required.

The phenomenon of haploinsufficiency (HI)—that is, growth phenotypes associated with the loss of function (e.g., deletion) of one allele in a diploid—is widespread amongst eukaryotes [7] and has been effectively applied in C. albicans to screen for genes involved in filamentous growth [8]. HI has been studied extensively in S. cerevisiae and offers a way to exploit the diploidy of C. albicans. While only ∼3% of the S. cerevisiae genome displays HI under the standard growth conditions [9], chemically induced HI is more specific. It has been shown in an assay termed the fitness test (also referred to as haploinsufficient phenotype assay) that target-specific inhibitory molecules typically induce a growth disadvantage (i.e., hypersensitivity) of heterozygous deletion strains corresponding to the drug targets and/or other mechanism of action (MOA)–related genes [10–14]. If this specificity is also prevalent in *C. albicans,* chemically induced HI by mechanistically characterized inhibitors could be used to identify essential cellular processes and genetic interactions relevant to this pathogen. Conversely, when a novel inhibitory compound is tested, the response (hypersensitivity and resistance; i.e., haploinsufficiency and haploproficiency, respectively) of specific heterozygous strains may provide phenotypic information reflecting the MOA of the compound.

**Figure 1:**
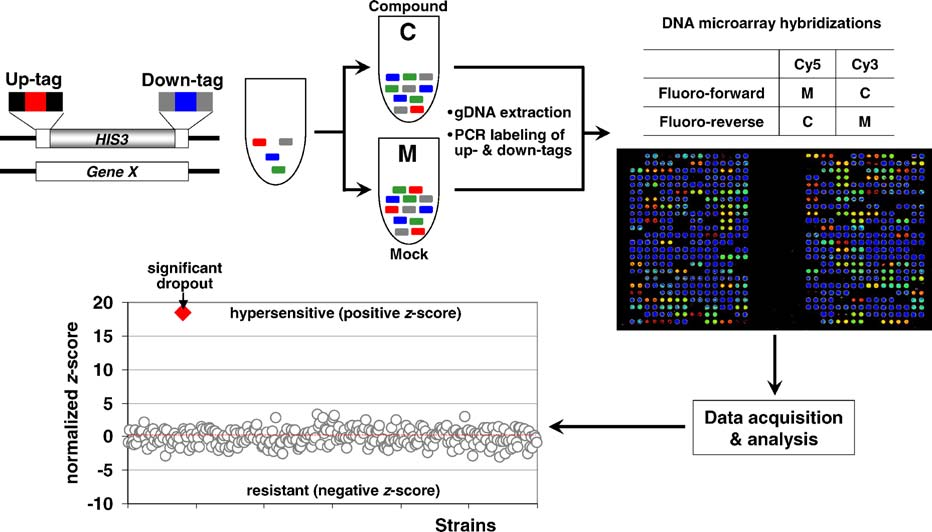
An Overview of the CaFT Individual heterozygous deletion strains contain two unique barcodes, up-tags (red) and down-tags (blue), flanked by two pairs of common primer sequences (black for all the up-tags, grey for all the down-tags) at the deleted allele. The CaFT pool contains 2,868 strains, representing ∼45% of the C. albicans genome. Aliquots of the pool are treated with an inhibitory compound (at different concentrations) or a mock treatment over 20 population doublings. The relative abundance of each strain is subsequently monitored by DNA microarrays competitively hybridized with amplified and labeled tags (using the common primer pairs) from the two treatments. The response of each strain to the compound is appraised by a normalized *z*-score, with a positive value indicating hypersensitivity, and a negative value relative resistance.

Here, we report on the application of chemically induced HI on a genomic scale in C. albicans. Drawing from analogous studies performed in *S. cerevisiae,* we term this assay the C. albicans fitness test, or CaFT (Figure 1). We systematically constructed 2,868 heterozygous deletion strains in which two unique barcodes were introduced in the up- and downstream regions of the deleted allele. Gene-specific barcodes differentiate heterozygotes from one another and enable their multiplex screening for HI growth phenotypes when challenged with antifungal agents. To readout growth changes resulting from drug treatment, each unique tag is amplified by PCR using common flanking primer sequences, labeled, and hybridized to a DNA microarray that identifies all barcodes (Figure 1). Statistical analyses enable identification of strains significantly affected in growth rate. With a CaFT of ∼45% genome coverage, we have examined mechanistically diverse inhibitory compounds with three objectives: 1) to determine and validate the prevalence and specificity of chemically induced HI in the pathogen, 2) to provide experimental data to functionally annotate genes and their genetic interactions involved in the cellular processes inhibited by these compounds, and 3) to establish the utility of the CaFT in MOA studies of inhibitory compounds for antifungal drug discovery.

## Results

C. albicans genes selected for construction of heterozygous deletion strains in this pilot study were chosen based on 1) their predicted orthologs being essential in *S. cerevisiae,* 2) their broad conservation across fungi (including Aspergillus fumigatus), and/or 3) sharing strong homology to genes conserved in higher eukaryotes. Approximately 29% of the C. albicans genes used are essential for viability [6], and Table S1 lists all genes examined in this study. Heterozygous deletion strains were constructed using previously described PCR methodologies [6]. A pool containing equal proportions of all 2,868 strains was prepared and aliquots frozen, with thawed aliquots used to perform all the CaFT experiments described. To identify specific haploinsufficiency and/or haploproficiency, a normalized *z*-score was used to assess the response of individual strains to inhibitory compounds (see Materials and Methods for details). For each strain, the normalized *z*-scores of both barcodes are determined by 1) the average (“historical”) behavior of this strain, as determined by each barcode, in a set of reference experiments with chemically diverse compounds (i.e., barcode-specific error modeling), and 2) the overall responsiveness of all the strains in a given experiment. Since the up- and down-barcodes are analyzed separately, individual strains are independently appraised twice in each experiment. A positive normalized *z*-score indicates a relative decrease in abundance in the compound-treated culture (i.e., insufficiency or hypersensitivity) and a negative normalized *z*-score indicates a relative increase (i.e., proficiency or resistance). To fully examine the effects elicited by any compound, experiments were performed at multiple sub-lethal inhibitory concentrations (ICs). A series of known antifungal agents with well-characterized MOAs were tested to validate the CaFT. Several of these compounds have been previously examined in the S. cerevisiae fitness test (ScFT), and they enable classification of functionally orthologous genes between organisms.

### CaFT Profiling of Inhibitors of Ergosterol Biosynthesis

**Figure 2:**
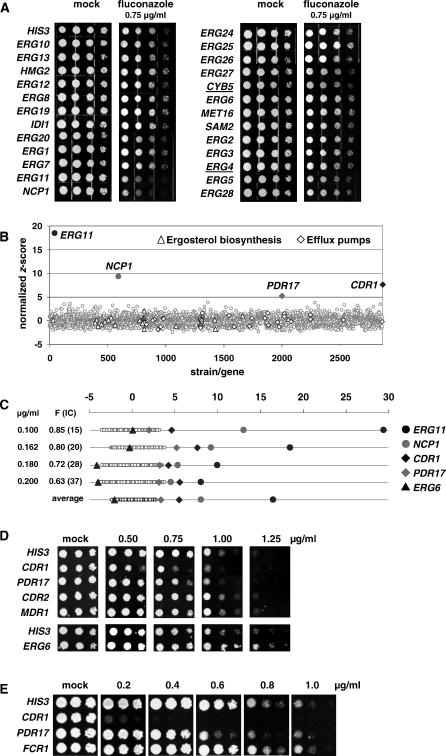
Characterization of Fluconazole-Induced HI by Spot Tests and CaFT Profiling (A) Specificity of chemically induced HI by fluconazole as determined by spot tests. The heterozygous deletion strains corresponding to genes involved in the ergosterol biosynthesis pathway were tested against fluconazole at 0.75 μg/ml. Note that the two underlined strains, *CYB5 (orf19.7049)* and *ERG4 (orf19.5379),* were not present in the CaFT pool and that an *ERG9 (orf19.3616)* strain was not constructed. The *HIS3* strain is used throughout this study as the wild-type control, since the *HIS3* gene, as an auxotrophic marker, was used to construct all the heterozygous deletion strains (see Materials and Methods). (B) CaFT profile of fluconazole at 0.162 μg/ml (with fitness, F, value of 0.80; i.e., IC_20_). The *x*-axis represents the strains in the CaFT pool, and *y*-axis the normalized *z*-scores of the corresponding strain in this experiment. (Although the *z*-scores of both up- and down-barcodes are assessed independently for each strain, the higher of the two was selected for display.) Significant outliers are highlighted by filled symbols, as their *z*-scores deviate significantly from the population. The *z*-scores of two groups of strains, corresponding to other genes involved in ergosterol biosynthesis or potential efflux pumps, are highlighted by open symbols. (C) CaFT profiles of fluconazole at different concentrations, with corresponding F values and ICs (in parentheses). In order to simplify a given CaFT profile, all the *z*-scores are displayed one-dimensionally according to the values but regardless of strain identities. Selected strains are marked so that their *z*-scores can be compared with the population and between experiments. Note that clotrimazole-induced HI of *CDR1* and *PDR16* was independently observed in the ScFT [13]. (D) Spot tests of heterozygous deletion strains identified in the CaFT *(CDR1, PDR17,* and *ERG6)* and the *CDR2* and *MDR1* strains (for their relevance, see the text) against multiple fluconazole concentrations. (E) Spot tests of homozygous deletion strains against fluconazole. (The *orf19* designations are as follows: *ERG11* = *orf19.922, NCP1* = *orf19.2672, CDR1* = *orf19.6000, PDR17* = *orf19.5839, ERG6* = *orf19.1631, CDR2* = *orf19.5958, MDR1* = *orf19.5604, FCR1* = *orf19.6817.*)

The CaFT strain pool includes heterozygotes for all but three genes involved in the ergosterol biosynthetic pathway (Figure 2A). This pathway represents a well-characterized target for antifungal agents (for a review see [15]) and provides an opportunity to determine the specificity of chemically induced HI in C. albicans by conventional methods (spot tests) and the CaFT.

Fluconazole is clinically used to treat C. albicans infections. It inhibits the sterol 14α-demethylase, which is encoded by *ERG11*. (Note that we use standard gene names as appear in the *Candida* Genome Database and MycoPathPD, with *orf19* designations given in the appropriate figure legends, and that the prefix *Sc* is used to refer to S. cerevisiae genes.) The heterozygous deletion strains for genes involved in ergosterol biosynthesis were tested against fluconazole by spot tests at multiple concentrations. While none showed significant HI under the standard growth conditions, the *ERG11* and *NCP1* (encoding a NADP-cytochrome P450 reductase required for the Erg11p function) strains displayed specific hypersensitivity to fluconazole (Figure 2A). Similarly, both strains showed fluconazole-induced HI in the CaFT over a wide range of ICs, as their *z*-scores were significantly differentiated from the population (Figure 2B and 2C; a summary of all the CaFT results described is provided in Table S1.) Two additional hypersensitive strains detected are *CDR1* and *PDR17* (orthologous genes in S. cerevisiae are *ScPDR5* and *ScPDR16,* respectively; note that the other ortholog of *ScPDR16, orf19.1027,* was not present in the pool). In addition, the *ERG6* strain showed relative resistance as indicated by the significant negative *z*-scores at higher concentrations (Figure 2C). The responses of these strains to fluconazole were confirmed by the spot tests (Figure 2D). Although overexpression of efflux pump genes *(CDR1, CDR2,* and *MDR1)* and *PDR16* (encoding a phosphatidylinositol transfer protein) was detected in fluconazole-resistant clinical isolates, only *CDR1* and *PDR16* have been correlated directly to resistance [16,17]. The strains for *CDR2* and *MDR1* showed no hypersensitivity to fluconazole in the CaFT (Table S1) or spot tests (Figure 2D), whereas homozygous deletion strains for *CDR1* and *PDR17* were markedly more susceptible to fluconazole (Figure 2E).

The five strains identified with fluconazole in the CaFT represent different aspects of its MOA: the drug target (Erg11p) and its accessory protein (Ncp1p), the principal efflux pump (Cdr1p), and two additional factors, Pdr17p and Erg6p, that are likely involved in drug uptake [18,19]. Another triazole (voriconazole) and imidazoles (ketoconazole, clotrimazole, econazole, and sulconazole) yielded similar CaFT profiles (Figure S1). The profiles of additional inhibitors of ergosterol biosynthesis, including terbinafine, lovastatin, and dyclonine, are described in Text S1 (Figure S2 and Table S1), and their results largely corroborate those determined in the ScFT [13]. Inhibitors that do not act through specific protein targets were also examined, including amphotericin B, which binds preferentially to ergosterol in the plasma membrane of fungi, and two toxic ergosterol analogs (ECC69 and ECC1384). In each case, complex CaFT profiles affecting multiple aspects of metabolism and membrane-related functions were produced but with no clear target(s) resolved (Figure S3).

### CaFT Profiling of Inhibitors of Other Enzymes and Protein Complexes

The CaFT profiles of enzyme inhibitors are usually concise. For example, *ALG7* and *AUR1* heterozygotes correspond to the target gene of tunicamycin and aureobasidin A, respectively, and each displayed highly significant and specific hypersensitivity to its cognate inhibitor (Figure S4). Similarly, the catalytic subunit *(FKS1)* and the regulatory subunit *(RHO1)* of glucan synthase were identified in the CaFT with their cognate inhibitors, caspofungin and ergokonin A (Figure S5). Brefeldin A is unusual in that it binds to the interface of two proteins, both of which are members of two protein families in C. albicans. Despite such apparent complexity, a single target pair, *SEC7* and *ARF2,* was robustly identified in the CaFT (Figure S6).

**Figure 3:**
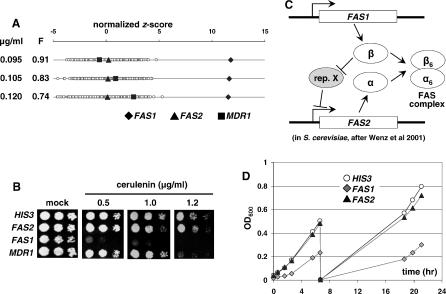
CaFT Profiling and Characterization of Cerulenin-Induced HI (A) CaFT profiles of cerulenin, with highlighted strains as follows: *FAS1 (orf19.979), FAS2 (orf19.5949),* and *MDR1 (orf19.5604)*. A homozygous deletion of *MDR1* is hypersensitive to cerulenin [21]; however, the heterozygous deletion strain showed no specific hypersensitivity at the ICs tested in the CaFT. (B) The spot tests of selected heterozygous deletion strains against cerulenin. Note that the hypersensitivity of the *MDR1* strain was only seen at the highest concentration of cerulenin tested. (C) A model for regulating stoichiometry of the FAS complex in S. cerevisiae. As shown by Wenz et al. [20], the expression of *ScFAS2* is repressed by an unknown transcription repressor (rep. X), which is in turn derepressed by free β subunit (Fas1p). According to this model, *ScFAS2* expression is dependent on free Fas1p to control the normal stoichiometry of the FAS complex. A similar regulatory mechanism in C. albicans may explain the observed cerulenin-induced HI of *FAS1* but not *FAS2* (see text for details). (D) *FAS1* is haploinsufficient under the standard growth conditions. In order to determine HI, cultures of the selected strains were first incubated for 6 h (reaching exponential growth) and then diluted to an OD_600_ of 0.005. The fresh cultures were incubated for another 12 h, after which the OD was monitored for an additional 4 h.

Cerulenin specifically inhibits the condensation reaction associated with the α subunit of the fatty acid synthase (FAS), a heteromultimeric complex of α (Fas2p) and β (Fas1p) subunits. Although not examined in the ScFT, cerulenin elicited reproducibly specific hypersensitivity of the *FAS1* strain but not of *FAS2,* even at the highest drug concentration tested (Figure 3A). These results were also demonstrated by spot tests (Figure 3B). In *S. cerevisiae,* the expression of *ScFAS2* is regulated in an ScFas1p-dependent manner to control the stoichiometry of the FAS complex [20]. Our results suggest that a similar regulatory mechanism exists in *C. albicans,* with the level of Fas1p being the critical factor controlling the FAS complex (see Figure 3C and Discussion). Consistent with this model, only *FAS1* exhibits HI under the standard growth conditions (Figure 3B and 3D).

Failure to detect hypersensitivity of the *FAS2* heterozygote to cerulenin reflects a more general difficulty in correctly identifying chemically induced HI within protein complexes, due to regulation of subunit stoichiometry, its assembly, or activation. To further investigate these potential issues, additional reference compounds that inhibit distinct protein complexes were examined.

**Figure 4:**
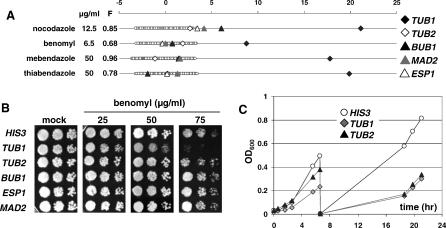
CaFT Profiles of Microtubule Inhibitors (A) CaFT profiles of nocodazole, benomyl, mebendazole and thiabendazole. Note that the ScFT experiments with these compounds have not been reported. Highlighted, in addition to *TUB1 (orf19.7308)* and *TUB2 (orf19.6034),* are *BUB1 (orf19.2678), MAD2 (orf19.1040)* and *ESP1 (orf19.3356)* strains that showed modest but reproducible hypersensitivity to nocodazole. The corresponding proteins are involved in mitotic checkpoint regulation and spindle pole body assembly [27]. Their relevance becomes apparent in the results described in Figure 7. (B) Spot tests of heterozygous deletion strains highlighted in (A) against benomyl. Note the lack of hypersensitivity of *TUB2* in spot tests is consistent with the CaFT results, implying that the failure to detect hypersensitivity of the *TUB2* strain in the CaFT is not due to poor performance of the barcodes. (C) *TUB1* and *TUB2* strains are haploinsufficient under the standard growth conditions (see Figure 3 legend for experimental details).

Microtubules are comprised of α- and β-tubulin subunits encoded by *TUB1* and *TUB2,* respectively. A potential binding site for benomyl, a microtubule depolymerizing agent, has been suggested in the core of S. cerevisiae β-tubulin [22], and the heterozygous deletion strains for both *ScTUB1* and *ScTUB2* are benomyl hypersensitive [23]. In the CaFT, however, only the *TUB1* strain displayed significant hypersensitivity to benomyl, as well as to additional microtubule inhibitors, including nocodazole, mebendazole, and thiabendazole, while the *TUB2* strain was marginally hypersensitive only to nocodazole (Figure 4A and unpublished data; note that *TUB4, orf19.1238,* was not represented in the CaFT). Spot tests confirmed the hypersensitivity of the *TUB1* strain and the lack of benomyl-induced HI for *TUB2* (Figure 4B). The lack of specific hypersensitivity of the *TUB2* heterozygote to most, if not all, of the microtubule inhibitors tested raises the question of how the stoichiometry of α- and β-tubulin subunits is regulated in C. albicans. In yeast, overexpression of *ScTUB2* results in lethality, whereas overexpression of *ScTUB1* does not [24]. Moreover, mutations in *ScTUB1* and *ScTUB2* are known for their unlinked noncomplementation [25]. In contrast, only an *ScTUB1* heterozygote is reported to be defective under the standard growth conditions [23,26]. In *C. albicans,* heterozygosity of both *TUB1* and *TUB2* confers growth defects (Figure 4C). Although stoichiometric regulation of tubulins in C. albicans is unknown, it may confound the chemically induced HI phenotypes associated with *TUB1* and *TUB2*.

**Figure 5:**
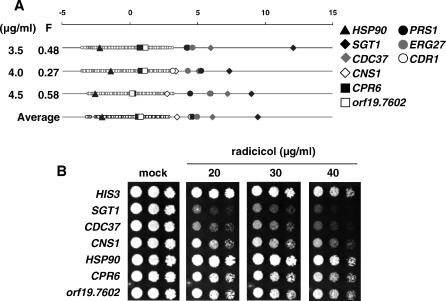
CaFT Profiles and Spot Tests of Radicicol (A) CaFT profiles of radicicol with genes encoding co-chaperones of Hsp90p highlighted, including *SGT1 (orf19.4089), CDC37 (orf19.5531), CNS1 (orf19.6052), orf19.7602* (an ortholog of *ScAHA1*), and *CPR6 (orf19.7654)* ([30] and references therein). Radicicol also elicited HI of *ERG27 (orf19.3240), CDR1* (*orf19.6000,* an efflux pump), and *PRS1* (*orf19.1575,* involved in cell integrity/stress signaling as reported [32]). (B) Spot tests of radicicol against heterozygous deletion strains corresponding to *HSP90* and its co-chaperones.

Radicicol inhibits cell growth by competitively binding to the conserved chaperone, HSP90 [28], a key molecular chaperone that, together with its co-chaperones, facilitates proper folding of multiple client proteins [29]. Unlike *S. cerevisiae,* which contains two HSP90 proteins, ScHsc82p and ScHsp82p, C. albicans possesses only one. Despite sharing all the conserved amino acid residues in both yeast HSP90 proteins implicated in radicicol binding [28], the *HSP90* heterozygous deletion strain lacked detectable hypersensitivity when tested in the CaFT (Figure 5A) or when examined either by spot tests (Figure 5B) or liquid IC determination (unpublished data). Instead, three *(SGT1, CDC37,* and *CNS1)* of the five potential Hsp90p-associated co-chaperones (*CPR6* and *orf19.7602* are also represented in the CaFT) displayed HI to radicicol (Figure 5A and 5B). Interestingly, the three yeast orthologs, ScSgt1p, ScCdc37p, and ScCns1p, all physically and genetically interact with ScHsc82p and/or ScHsp82p [30,31]. Although radicicol-induced HI of *HSP90* was not detected, the CaFT profiles reflected other aspect of the target; that is, activation of Hsp90p by its co-chaperones (see Discussion).

Additional inhibitors of protein complexes tested with informative mechanistic CaFT profiles included cytochalasin D, roridin A, and verrucarin A. Cytochalasin D inhibits both association and dissociation of actin filaments in vitro. The CaFT results reveal a particular aspect of actin polymerization affected in vivo by cytochalasin D, namely, the branching of actin cables to produce cortical actin, as multiple members of the ARP2/3 complex showed markedly HI (Figure S7). The structurally related mycotoxins roridin A and verrucarin A both noticeably affected multiple subunits of the initiation factor eIF3 complex (Figure S8).

### CaFT Profiling and MOA Studies of 5-Fluorouracil, 5-Fluorocytosine, and Tubercidin

**Figure 6:**
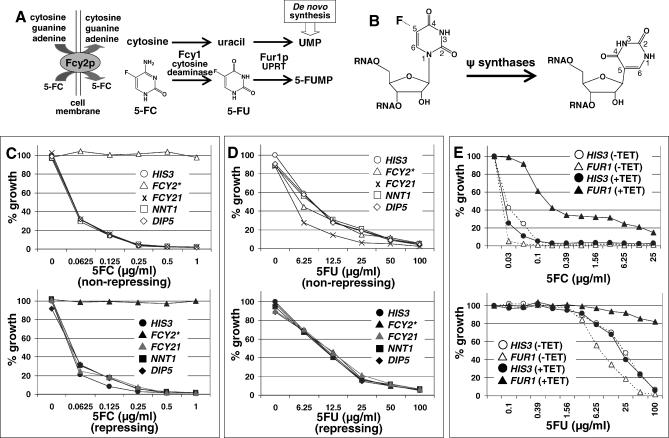
Genetic Characterization of 5-FC and 5-FU in C. albicans (A) Pyrimidine salvage pathway, and transport and metabolism of 5-FC (see text for details). (B) A hypothesis that fluorinated uracil blocks the formation of pseudouridine in rRNA. Note that pseudouridines are a prerequisite for ribosomal RNA processing [34]. (C and D) Functional characterization of potential permeases or transporters involved in the uptake of 5-FC (C) and 5-FU (D). A homozygous deletion strain for *FCY2* (distinguished by the asterisk) and conditional shut-off (tetracycline repressible promoter, GRACE) strains [6] for genes indicated were tested in the absence (i.e., the non-repressing conditions, top panels) or the presence (i.e., the repressing conditions, bottom panels) of 100 μg/ml tetracycline against both compounds at the concentrations indicated. Note that 1) in (C) the *FCY2* homozygous deletion strain was resistant to 5-FC, and 2) in (D) only the *FCY21* GRACE strain exhibited slightly increased susceptibility to 5-FU under the non-repressing condition, suggesting that overexpression of *FCY21* (from the tetracycline promoter, as observed in other cases, unpublished observations) may marginally facilitate the uptake of 5-FU. (E) Suppression of 5-FC (top) and 5-FU (bottom) antifungal activity by genetic depletion of Fur1p. The *FUR1* and *HIS3* (the control) GRACE strains were grown in the presence (100 μg/ml, +TET) or absence (−TET) of tetracycline with 5-FC or 5-FU at the concentrations indicated. Individual growth was normalized with growth of the *HIS3* strain without antifungal drug in the presence or absence of tetracycline. Note that when the expression of *FUR1* was repressed, the strain was markedly resistant to both compounds, whereas it was modestly hypersensitive under the non-repressing conditions, likely due to overexpression of *FUR1* from the tetracycline promoter. (The *orf19* designations are as follows: *FCY2* = *orf19.1357, FCY21* = *orf19.333, NNT1* = *orf19.4118, DIP5* = *orf19.2445,* and *FUR1* = *orf19.2640.*)

The preceding examples demonstrate the specificity of chemically induced HI and the biological relevance of information contained within CaFT profiles; that is, small molecules that selectively inhibit proteins or protein complexes typically elicit specific CaFT profiles comprising the target proteins and/or other factors that functionally interact with the targets. The base analogs, 5-fluorocytosine (5-FC) and 5-fluorouracil (5-FU), on the other hand, do not exert inhibitory effects directly on specific proteins. We tested whether both analogs and tubercidin (7-deazaadenosine) elicit specific HI indicative of their MOAs. 5-FC and 5-FU are pro-drugs whose MOA has been well characterized in S. cerevisiae and to a lesser extent in C. albicans (see below). 5-FC, once inside the cell, is converted to 5-FU by cytosine deaminase, and 5-FU to 5-FUMP by uracil phosphoribosyltransferase (UPRT). Both enzymes are part of the pyrimidine salvage pathway (Figure 6A). 5-FU, in the ScFT, has been shown to induce HI of several genes involved in rRNA processing and ribosomal biogenesis [11,13]. In the CaFT, 5-FU, 5-FC, and tubercidin elicited responses of two distinct groups of heterozygous deletion strains, reflecting collectively a common MOA. While the majority of hypersensitive strains correspond to genes whose S. cerevisiae orthologs are involved in biogenesis of the 60S ribosomal subunit, resistant strains predominantly correspond to those encoding protein subunits of the U3 snoRNP complex, which is required for 18S rRNA processing (Figure S9). As ribosomal RNAs account for over 80% of the total cellular RNA, the preferential effects on rRNA biogenesis by these inhibitors may simply reflect the relative abundance of rRNAs. Another analog of uracil, 6-azauracil (6-AU, known to inhibit the IMP dehydrogenase involved in purine biosynthesis [33]), on the other hand, did not produce a ribosomal biogenesis–related profile (Figure S9A). The distinct CaFT profiles between 6-AU and 5-FC, 5-FU, or tubercidin suggest that a more specific mechanism may be involved (see Discussion).

The CaFT profiles of 5-FU and 5-FC also provide mechanistic insights into their uptake and metabolism. Counterintuitively, 5-FC is over 200-fold more potent than 5-FU (Figure 6C and 6D). The underlying mechanism for this difference is revealed in their CaFT profiles. *FCY2* encodes a permease for cytosine and purines. The corresponding heterozygous deletion strain displayed specific and significant resistance to 5-FC, but not to 5-FU or tubercidin (Figure S9A). A homozygous deletion of *FCY2* was highly and specifically resistant to 5-FC (Figures 6C, 6D, and S9B), suggesting a direct and specific role of Fcy2p in transporting 5-FC. In contrast, 5-FU may enter the cell by passive diffusion (Figure 6D), hence the relatively low potency. Similarly, the nucleoside transporter Nnt1p (absent in S. cerevisiae) was identified as responsible for the uptake of tubercidin (Figure S9B). Another notable resistant strain identified in the CaFT profiles of both 5-FC and 5-FU corresponds to *FUR1* (Figure S9A). *FUR1* encodes UPRT that converts both pro-drugs to 5-FUMP (Figure 6A). A loss of function of *FUR1* is expected to confer resistance to both 5-FC and 5-FU, as demonstrated by the high level resistance of a *FUR1* conditional shut-off strain under the repressing conditions (Figure 6E). These results predict specific resistance mechanisms that are known for 5-FC. Since the pyrimidine salvage pathway (Figure 6A) is not essential when the de novo pathway is intact, any loss-of-function mutations in genes involved in the former may have no effect on viability, and yet they may confer specific resistance, as demonstrated by mutations in the uptake and/or metabolism of this compound in S. cerevisiae (e.g., [35]) and, more importantly, in clinical isolates of C. albicans (e.g., [36,37]). This may also account for the primary resistance in *C. albicans;* that is, the inherent resistance to 5-FC without prior exposure, which is observed in ∼3% of clinical isolates [38].

### Novel Antifungal Compounds That Affect Microtubule Dynamics

**Figure 7:**
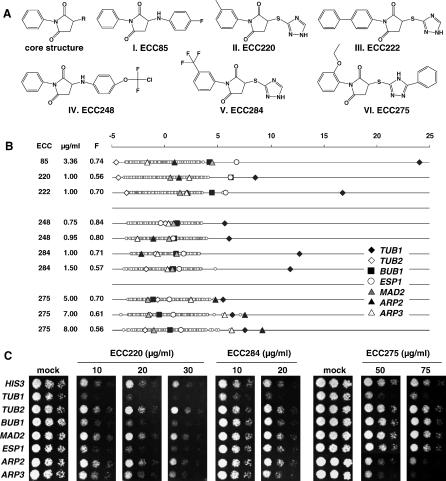
CaFT Profiling and Characterization of Novel Antifungal Compounds (A and B) Chemical structures (A) and the CaFT profiles (B) of novel and structurally related compounds that are predicted to affect microtubule dynamics. (C) Spot tests of representative compounds (ECC220, ECC284, and ECC275) confirm hypersensitivity detected in the CaFT. For *orf19* designation, see Figure 4.

Mechanisms inferred from screening known antifungal agents in the fitness tests of S. cerevisiae and C. albicans imply that MOA information could similarly be obtained for new compounds. To test this possibility, we examined a group of chemically related active compounds with unknown MOA (Figure 7A). CaFT profiles identified the *TUB1* strain as strikingly hypersensitive to all compounds examined (Figure 7B). Distinct secondary hypersensitivity, including heterozygotes compromised for mitotic checkpoint and cortical actin function, enabled compounds to be grouped into three sub-classes (Figure 7A and 7B). Selected compounds from each class were examined in spot tests, which largely confirmed the CaFT results (Figure 7C). Compared with known microtubule inhibitors (Figure 4), the CaFT profiling suggested that these compounds inhibit growth, likely by affecting the microtubule function.

**Figure 8:**
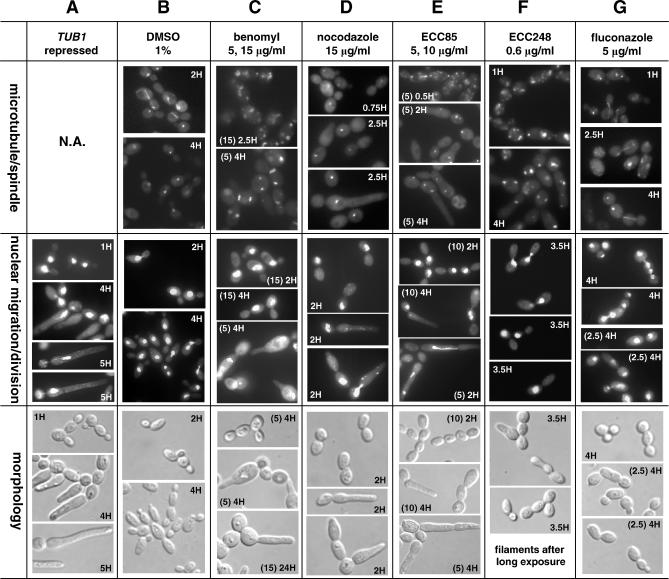
MOA Characterization of Compounds That Affect Microtubule Dynamics (A) The phenotypic defects associated with a *TUB1* conditional shut-off strain [6] examined under the repressing conditions (with time after the switch indicated on each photo). Under the non-repressing conditions, this strain was indistinguishable from the mock-treated wild-type cells (unpublished data). Nuclear migration was visualized by DAPI straining with morphology viewed under Nomarski optics. In (B–G), a strain carrying a Tub1p-GFP fusion was used. (B) The microtubule/spindle structures (as visualized by GFP-Tub1 fusion), nuclear migration/division, and morphology of the mock-treated cells. (C–G) The microtubule/spindle structures, nuclear migration, and morphology of cells treated with benomyl (C), nocodazole (D), ECC85 (E), ECC248 (F), and fluconazole (G). The concentrations (μg/ml, in parentheses) and incubation times are indicated on each photo.

To further characterize the MOA of these compounds, multiple microtubule-based secondary assays were performed. Microtubules form the core structural component of the nuclear mitotic spindle and are necessary for chromosomal movement. In the yeast, the movement of nuclei to the bud neck before mitosis and the subsequent separation of nuclei depend on cytoplasmic microtubules [39,40]. In the wild-type cells, these events occur in a highly coordinated cell cycle–dependent manner such that the large-budded cells are almost never observed without the nucleus at, or extended through, the bud neck. As nuclear migration defects associated with microtubule perturbation can readily be visualized by DAPI staining of DNA, we examined the terminal phenotypes associated with a *TUB1* conditional shut-off strain and compared them with those observed in the wild-type C. albicans cells chemically inhibited with benomyl, nocodazole, and representative compounds from each sub-class (ECC85, ECC248, and ECC275). One hour after *TUB1* repression, cell division and nuclear migration were largely arrested in the majority of the cells examined, as large-budded cells were predominantly observed, with nuclear staining being restricted to the mother cell (Figure 8A). To visualize microtubules, a C. albicans strain carrying a Tub1p-GFP fusion (under the *TUB1* promoter, otherwise indistinguishable from the wild-type parental strain) was used to compare the effects of known microtubule inhibitors versus these ECC compounds. In the mock-treated (1% DMSO) cells, two distinct sub-cellular microtubule structures were observed depending on the cell cycle: the mitotic spindles (visualized in the large-budded cells) and the spindle-pole bodies (visualized prominently in the small-budded cells) (Figure 8B). As expected, benomyl and nocodazole ablated the mitotic spindles, resulting in patches or aggregates of tubulin. Defects in nuclear division and G2 cell cycle arrest, as well as a more pronounced filamentous growth, were also observed (Figure 8C and 8D). These results are in agreement with the previous observations [41,42] and establish a strong phenotypic correlation between genetic depletion and chemical inhibition of the tubulin function. The defects caused by ECC85 (Figure 8E), ECC248 (Figure 8F), and ECC275 (unpublished data) were similar to those, described above, seen for cells treated with nocodazole, benomyl, or genetically depleted for *TUB1*. However, cells treated with the control compounds fluconazole (Figure 8G) and amphotericin B (unpublished data) lacked microtubule and/or cell cycle defects. To address whether the representative compounds of each ECC sub-class directly inhibit microtubule polymerization, their effects on in vitro bovine microtubule polymerization were examined. ECC85, ECC248, and ECC275 each displayed a dose-dependent inhibition of microtubule polymerization. Moreover, the IC50 (of V_max_) of ECC85 and ECC248 was approximately three times lower that of nocodazole (Figure S10). Collectively, these data provide in vitro and in vivo evidence that these compounds perturb the function of microtubules in C. albicans.

## Discussion

The impact of the baker's yeast, *S. cerevisiae,* as a eukaryotic model system in biology is immense. Indeed, the extensive knowledge base of biological information gained from these studies has greatly facilitated our understanding of fungal biology in medically significant pathogens, including C. albicans (e.g., [3,4,6,8]) and A. fumigatus (e.g., [43,44]). However, the differences in lifestyles and the functional disconcordance at the genomic level between yeast and fungi [6,8], combined with the extensive genome sequence information across the fungal kingdom that is now available [45–48], provide a timely opportunity to develop fungal genomics tools for basic and applied research in this field. The CaFT, based on the chemically induced responses of heterozygous deletion strains, exploits the diploid nature of C. albicans and provides a global genetic strategy to directly perform such studies in this pathogen.

The genetic basis of the fitness test is the consequence of heterozygosity in the presence of an inhibitory compound, and hence its readout is based on observable phenotypes and is often concise. As first reported in S. cerevisiae [11–13], chemically induced HI is largely restricted to the target and to other genes whose functions are genetically associated with the target and/or MOA of the inhibitor. This is in stark contrast with results of expression profiling of inhibitory compounds. Examination of the transcriptional responses of either S. cerevisiae or C. albicans to azoles revealed nearly 300 significantly responsive genes [49,50]. Although many genes in the ergosterol pathway are included in these complex profiles, there is a lack of clear quantitative correlation between the level of responsiveness and their biological relevance to the primary MOA of azoles. The CaFT profiles of fluconazole, however, contain only five significant responsive genes corresponding to the target (Erg11p), its accessory factor (Ncp1p), the principal efflux pump (Cdr1p), and two additional factors involved in drug uptake (Pdr17p and Erg6p, Figure 2). For most reference compounds examined, significantly responsive strains identified in the CaFT were validated independently, suggesting a low rate of false positives and the effectiveness of the statistical analyses employed.

Enzyme inhibitors such as azoles, terbinafine, dyclonine, tunicamycin, aureobasidin A, glucan synthase inhibitors, and brefeldin A all induce significant and specific hypersensitivity of heterozygotes corresponding to their targets (including co-factors and regulatory subunits) in the CaFT. Thus, a compelling indication of MOA is generally achieved. Cerulenin is an exception to this trend, as its known target, *FAS2,* showed no HI. Instead, the heterozygote for the other subunit of the FAS complex, *FAS1,* was noticeably hypersensitive. These results are consistent with the Fas1p-dependent stoichiometric regulation of the FAS complex demonstrated in S. cerevisiae [20]. We speculate that a *FAS1* strain is specifically hypersensitive to cerulenin because only in this strain is the overall level of the FAS complex compromised, whereas Fas2p levels in the *FAS2* heterozygote are likely upregulated by normal levels of Fas1p to restore the wild-type levels of the FAS complex (see Figure 3C for details).

Inhibitors of protein complexes, such as cytochalasin D (Figure S7), roridin A, and verrucarin A (Figure S8), typically produced CaFT profiles reflecting a rich diversity of biologically relevant responses of strains that corroborate their known MOAs. Despite this, discerning the specific subunit targeted within a complex is problematic, as multiple components of the complex are routinely observed as chemically haploinsufficient. Thus, CaFT profiling only yielded a general classification of MOA (ARP2/3-based cortical actin assembly and eIF3-directed translation, respectively), and further studies would be required to refine their molecular targets. Radicicol provides another cogent example of the limitation of the CaFT, in that its well-characterized target (Hsp90p) was unresponsive to drug treatment as a heterozygote (Figure 5). Instead, strains for its co-chaperones were identified as radicicol sensitive. These results are consistent with the observations that, in *S. cerevisiae,* the activity of Hsp90p is regulated at the post-transcriptional level; that is, it is activated by its co-chaperones [29]. Similar regulatory control of Hsp90p in C. albicans would account for our observations.

Mechanistic insights of fitness test profiling can also be extended to nucleoside and base analogs ([11,13] and this study). For example, both ScFT and CaFT profiling reinforce that ribosomal RNAs are likely the primary target on which the toxicity of 5-FU and 5-FC is exerted. It is unclear whether rRNA processing and ribosomal biogenesis is affected largely due to the relative abundance of rRNAs over other RNAs, or if these compounds exert a more specific mechanism targeting rRNAs (Figure 6B). Ribosomal RNAs are post-transcriptionally modified and contain a disproportionately high number of pseudouridines, an essential modification produced by isomerization of uridine via base rotation and subsequent linking of uracil to the sugar moiety at the 5 position of the pyrimidine ring (Figure 6B) [34]. Thus, rather than a bulk flow MOA for their preferential rRNA effects, a more specific ribosomal biogenesis defect may result from the incorporation of 5-fluorinated uridine into rRNAs, thus effectively blocking this base rotation for the formation of pseudouridine. Consistent with this view, 6-AU, which is able to undergo base rotation, did not yield a CaFT profile indicative of an rRNA processing MOA.

CaFT profiles often contain additional responsive genes, some of which illuminate drug uptake and efflux mechanisms that play important roles in acquired and inherent resistance to antifungal drugs such as azoles and 5-FC. For example, *CDR1* displays chemically induced HI against all the azoles we examined, as well as other predicted efflux substrates, including brefeldin A, as reported [21], radicicol (Figure 5A), cytochalasin D (Figure S7), PF1163A, and latrunculin A (unpublished data). Interestingly, the CaFT also identifies heterozygotes with a selective growth advantage (haploproficiency) in the presence of inhibitory compounds. This is best illustrated by the identification of the well-characterized resistance mechanisms for 5-FC/5-FU in the CaFT. Heterozygosity of the two genes involved in drug uptake *(FCY2)* and metabolic conversion *(FUR1)* [35–37] result in haploproficiency (Figures 6 and S9). A similar role was established for the uptake of tubercidin by Nnt1p (Figure S9). Other responsive genes may underscore their genetic interactions with the drug targets; for example, the dependence of mitotic checkpoint regulation and spindle pole body assembly on microtubule integrity (nocodazole, Figure 4A; ECC85, Figure 7B), and a possible stress response associated with tunicamycin (Figure S4B). Several inhibitors (radicicol, terbinafine, tunicamycin, aureobasidin A, and cytochalasin D) also induce hypersensitivity of heterozygotes whose biological relevance to the targets is unclear. Such hypersensitive strains may reflect potential synthetic interactions between chemical inhibition of the drug target and heterozygosity of the second gene. Alternatively, chemically induced HI in some cases may reflect potential secondary targets of these compounds, as previously suggested [13].

In this study, over 100 C. albicans genes have been classified according to their chemically induced HI with the ∼20 types of inhibitors tested. Although some of these genes have been experimentally characterized in *C. albicans,* most are annotated solely by their similarity to S. cerevisiae orthologs. Their phenotypes in response to selected inhibitors provide a first level of functional annotation. This is best exemplified by screening brefeldin A, which binds to the interface of two proteins (ARF and GEF), both of which are members of conserved protein families in C. albicans. CaFT profiling identified the functional pair that is most susceptible to brefeldin A (Figure S6). The CaFT is, however, currently biased against the identification of C. albicans specific genes for two reasons: 1) only ∼3% of the genes represented in this pilot study are *Candida*-specific (as defined by their absence of clear homologs in *S. cerevisiae, A. fumigatus, S. pombe,* or the human genes, using a BLAST cutoff of 1 × 10^−20^), and 2) the selected probe inhibitors have intrinsic activity against both S. cerevisiae and *C. albicans,* and are mechanistically conserved. It is therefore not surprising that the majority of genes identified in this study are conserved. Notwithstanding these restrictions, Nnt1p, a nucleoside transporter absent in *S. cerevisiae,* was identified as required for the uptake of tubercidin in C. albicans (Figure S9), suggesting that expanding genomic coverage in the CaFT and screening more diverse inhibitory compounds will likely uncover specific features of C. albicans biology.

Other potential limitations to the CaFT include: 1) The C. albicans diploid genome contains allelic polymorphisms, some of which may inactivate genes and confound their identity as haplo-responsive strains. However, as summarized in Table S2, failure to detect hypersensitivity of strains for target genes (e.g., *FAS2, HSP90*) was not due to allelic differences, which is only rarely observed amongst 69 other genes pertinent to the CaFT profiles reported here. Although eight of these genes possess some allelic differences, they result in only one or two conservative amino acid change(s) in their corresponding proteins. 2) The inherent HI under the standard growth conditions, as demonstrated by *TIF35* (Figure S8), may obscure chemically induced HI. However, severe intrinsic HI is rarely observed. Other heterozygotes with modest growth defects (e.g., *FAS1, TUB1*) are not problematic to assay in the fitness test format. 3) The detection of strain responses in the CaFT relies solely on robust hybridization signals of error-free barcodes. The introduction of double barcodes significantly reduces the occurrence of unassayable strains (e.g., [14]). Barcodes of such strains can be sequenced and microarrays redesigned with fully complementary oligonucleotides (e.g., [51]), or such strains can be simply reconstructed with new barcodes. 4) In *S. cerevisiae,* it is known that deletion strains can become aneuploid [52]. Our strain stocks are stored with minimal manipulation, and the strain pool is preserved in aliquots. All the CaFT experiments were performed with aliquots from the same preparation. Although the problem of aneuploidy has not been examined in *C. albicans,* our standard practices should minimize its occurrence.

We have also explored the CaFT assay as an approach to predicting the MOA of novel antifungal compounds. To this end, a collection of structurally related synthetic compounds with antifungal activity but unknown MOA were examined. Their profiles were highly related to known microtubule inhibitors and highlighted by marked hypersensitivity of the *TUB1* heterozygote (Figures 4 and 7). Subtle differences in CaFT profiles between these compounds were observed, most notably the hypersensitivity of *BUB1* and *ESP1* to one compound (ECC220), as well as a potential secondary effect on cortical actin by a second compound (ECC275). These differences may reflect different structure–activity relationships and/or off-target effects between structurally related compounds, as similarly demonstrated between fenpropomorph and related compounds in the ScFT studies [11]. The primary MOA of these ECC compounds as microtubule inhibitors was verified genetically (Figure 8) and biochemically (Figure S10). First, the representative ECC compounds phenocopy the genetic depletion and the chemical inhibition of *TUB1;* that is, they promote pronounced cell cycle arrest and nuclear migration defects at early time points and a subsequent pseudohyphal morphology at later time points, as reported with nocodazole-treated C. albicans [41]. Second, Tub1p-GFP sub-cellular structure studies reveal that these ECC compounds, nocodazole, and benomyl all similarly disrupt microtubule structures. Third, these ECC compounds inhibit in vitro microtubule polymerization, indicating their primary MOA as microtubule inhibitors.

CaFT screening of inhibitory compounds, combined with recent target validation strategies in both C. albicans [6,53] and A. fumigatus [43,54], may provide several significant advantages to antifungal drug discovery. The CaFT facilitates a reverse genetic approach; that is, it links traits (responses to inhibitory compounds) to preexisting mutations (heterozygous deletion strains), potentially on a global scale and within the major fungal pathogen. Drug resistance mechanisms can be identified early and in parallel to MOA determination of potential antifungal agents. Drug targets are identified empirically and are biased towards those with intrinsic susceptibility to chemical inhibition. Moreover, only subsequent to the identification of a target–inhibitor interaction is target validation in key fungal pathogens required [6,43,53,54]. In this way, compound–target pairs may be efficiently prioritized as antifungal drug leads according to their chemical attributes, MOA, and target validation information. In summary, an assayable and comprehensive target set screened across broad chemical diversity may offer a new opportunity to identify antifungal agents that are both mechanistically and structurally novel.

## Materials and Methods

### Genome annotation.

The C. albicans genome sequence at 10.9X coverage was determined by the Stanford Genome Technology Center (http://www-sequence.stanford.edu/). A precise genome annotation for C. albicans [3] was not publicly available during the course of this project. Instead, a list of 7,680 open reading frames (ORFs) encoding proteins ≥100 amino acids provided in an earlier release was used to initiate an internal annotation effort. To select ORFs for construction of heterozygous deletion strains, only those satisfying either of the following conditions were initially chosen: 1) ORFs (*n* = 4,068) with clear homologs (BLAST e-value < 1 × 10^−20^) at amino acid level in other fungal species, or 2) ORFs (*n* = 1,635) with no clear fungal homolog but ≥ 600 nucleotides in length. Recent S. cerevisiae annotation efforts demonstrate that such rules provide 99% and 98% confidence of a bona fide gene locus rather than a spurious ORF [5,55]. The high degree of conservation in gene structure between S. cerevisiae and *C. albicans,* including average length, intron structure, intron-occurring frequency, GC-contents, and promoter elements [3], strongly reinforces the applicability of such gene-coding “rules” to C. albicans genome annotations.

### C. albicans heterozygote strain construction and the CaFT strain pool composition.

Heterozygous deletion strains were constructed essentially as in *S. cerevisiae,* with some modifications [6,56,57]. Briefly, individual strains were constructed by replacing the entire ORF of a particular gene with a cassette of *HIS3* gene flanked by distinct upstream and downstream barcodes, termed up-tag and down-tag, respectively. Each tag is in turn flanked by two primer sequences that are common to all the up- or down-tags (Figure 1). The nucleotide sequences for up- and down-tags, as well as the flanking common primers, were as used in the S. cerevisiae deletion project [57]. This configuration provided two very powerful features to these double-barcoded heterozygote strains: 1) it enables PCR amplification of large numbers of distinct up- or down-tags using two sets of common PCR primers, and 2) it allows the identification of each heterozygote strain (and the corresponding gene) via the identities (i.e., the sequences) of a unique pair of up- and down-tags. More specifically, PCR amplification of a deletion cassette containing a *HIS3* auxotrophic marker was performed using 99-nucleotide (nt) oligos containing 1) 5′ sequence of 43 nt directing homologous recombination at the 5′ or 3′ of the target gene, 2) internal strain-identifying unique 20-nt barcodes and flanking common primer sequences for the ultimate PCR amplification of the barcodes in the fitness test assay, and 3) 18 nt of 3′ sequence, which anneals to the 5′ or 3′ of the *CaHIS3* gene and facilitates PCR amplification of the disruption cassette for transformation. Transformants were genotyped for correct integration into the target locus by PCR to confirm the expected 5′ and 3′ junctions. Genes chosen for disruption were selected based on prior knowledge that their closest homolog is 1) known to be essential in *S. cerevisiae,* 2) a member of a gene family known to display a growth phenotype when deleted in *S. cerevisiae,* or 3) conserved in A. fumigatus or other fungal organisms. In total, this procedure was applied to 2,868 distinct genes, which represented approximately 45% of the C. albicans genome. They included all the experimentally demonstrated essential genes we reported previously [6]. These 2,868 heterozygote strains were cultured individually and manually mixed in approximately equal proportions to generate a pool of C. albicans strains, aliquots of which were frozen and used in all the experiments. Although the full collection of strains comprising the CaFT is not available at this time, individual strains pertinent to the profiles presented in this study are accessible to investigators, following the standard Merck Material Transfer Agreement and clearance procedures.

### Construction of homozygous deletion strains.

Heterozygous deletion strains were used to construct homozygous deletions with the PCR-based method described [6], except that the coding region of the second allele was replaced by the *SAT-1* gene, which confers resistance to nourseothricin.

### Antifungal compounds.

Compounds were purchased from Sigma-Aldrich (http://www.sigmaaldrich.com/), with the following exceptions: caspofungin from Merck (http://www.merck.com/), fluconazole from Pfizer (http://www.pfizer.com/), and aureobasidin A from Takara/Fisher (http://www.fishersci.com/). ECC220 is available from AKos Consulting and Solutions GmbH (Basel, Switzerland; http://www.akosgmbh.eu/), ECC22 from Interchim (Montlucon, France; http://www.interchim.com/), ECC248 from ASINEX (Moscow, Russia; http://www.asinex.com/), and ECC275 from Ambinter (Paris, France; http://www.ambinter.com/).

### The CaFT DNA microarrays.

We custom-built a set of two DNA microarrays using Amersham CodeLink Activated Slides (http://www.gelifesciences.com/). These microarrays contain DNA oligos identical to the up- or down-tags, with each oligonucleotide duplicated side by side. Each array contains 16 sub-arrays (of 16 × 24 spots), for a total of 3,072 duplicated features, corresponding to all the strains in the CaFT pool, and various controls.

### The CaFT experiments and data analysis.

For each compound tested, a prior IC curve was determined using the CaFT strain pool (with an initial OD_600_ of 0.025) in liquid RPMI medium (in 96-well format), grown at 30 °C for 15 h. Based on the IC curve, 5-ml cultures of the CaFT pool (with the initial OD_600_ of 0.025) were treated with the selected compound at multiple concentrations, together with mocks. After 15 h of growth at 30 °C, the fitness (F) values of compound-treated cultures were determined, F = OD_(compound-treated)_ / OD_(mock)_; that is, the inverse of IC. Cultures of desired F values were selected and diluted to OD_600_ 0.05 with the medium containing the compound at the original concentrations. After another 23 h of growth, all cultures were collected and cell pellets frozen. Following extraction (MasterPure Yeast DNA Purification Kit; Epicentre, http://www.epibio.com/), genomic DNA preparations from compound-treated and mock cultures were PCR amplified with Cy3- or Cy5-labeled common primers. Labeled tags from compound-treated and mock cultures were mixed and hybridized against the corresponding DNA microarray (Figure 1). (The up- and down-tags were amplified and hybridized separately.) The intensities of signal were obtained for each barcode of compound-treated and mock cultures from a single DNA microarray. They were first converted to a log_2_ scale before further analysis. For each tag, the log-fold-dropout (LFD) was computed as the average differences between the mock and the compound-treated. Following the compilation of results for 57 diverse compounds (referred to as the reference set), a statistical profile was computed for each tag by modeling the distribution of LFD as a mixture of a normal component (random variation of the tag when the strain growth is not preferentially affected by the compound) and a uniform distribution (representing unusual LFDs for a given tag). This model was optimized using the EM algorithm [58], and the parameters of the normal component were stored as a model of the intrinsic variability of a given tag through the complete experiment. For any tag in any given experiment, the computed LFDs are converted to a *z*-score by using the parameters of the corresponding tag profile. To facilitate the comparison of experiments with different LFDs distribution, we applied a multiplicative correction factor to the LFDs that corresponds to 1/*σ_z_*, where *σ_z_* is the standard deviation of the *z*-scores for that experiment. The *z*-scores were then recomputed from these normalized LFDs and are referred to as normalized *z*-scores. To avoid result overinterpretation, the *z*-scores are not converted to *p*-values and are considered the final quantitative result of the experiment. This approach has the advantage of accounting for the nonspecific sensitivity of some tags/strains to chemical insults and for unexpectedly variable tags/strains, and thus greatly reduces the frequency of false positives. Contrary to other approaches (e.g., [59]), our analyses do not rely on a correlation between signal variability and signal strength, but rather are modeled on a tag-by-tag basis, taking advantage of multiple experiments as pseudo-replicates, and rely on the EM algorithm to identify outliers. We hold that using a set of compound-treated cultures to establish a statistical model is a superior approach to using a set of replicated mock cultures since the latter would not model nonspecific responses to variations such as growth conditions and chemical insult (e.g., broadly hypersensitive stains).

### C. albicans spot tests.

Individual heterozygous strains were first grown in liquid medium (without compound) to OD_600_ ∼2. Cultures were diluted to OD_600_ 0.2 and transferred to 96-well microtiter plate, followed by 1:5 serial dilutions. Aliquots (3 μl) of diluted cultures were spotted onto solid media (YPD) containing 1% DMSO (mock) and inhibitory compounds at the concentrations indicated with 1% DMSO. Plates were incubated at 30 °C, and photographed after 2 d (unless otherwise noted in the figure).

### In vitro tubulin polymerization assay.

Cytoskeleton CytoDYNAMIXTM Screen 3 (http://www.cytoskeleton.com/) was employed in these assays, in which purified tubulin (>99% pure tubulin isolated from bovine brain, from Cytoskeleton catalog # TL238) was used to follow the in vitro microtubule polymerization process. To initiate the assay, different concentrations of ECC85, ECC248, ECC275, or nocodazole were added to the standard assay solution, which contains 3.5 mg/ml purified bovine tubulin in 80 mM PIPES (pH 6.9), 1 mM MgCl_2_, 1 mM EGTA, 1 mM GTP, 5% glycerol, and 1% DMSO. The assay mixtures were incubated at 37 °C for 60 min, and the polymerization process was monitored by turbidity measurements at OD_340_ at 1-min intervals.

## Supporting Information

Figure S1 CaFT Profiles of Imidazoles and a Triazole (222 KB PPT)

Figure S2 CaFT Profiles of Inhibitors of Enzymes Involved in Ergosterol Biosynthesis (389 KB PPT)

Figure S3 CaFT Profiles of Amphotericin B and Two Ergosterol Analogs (189 KB PPT)

Figure S4 CaFT Profiles of Tunicamycin and Aureobasidin (2.4 MB PPT)

Figure S5 CaFT Profiles of the β-1,3-glucan Synthase Inhibitors, Caspofungin and Ergokonin A (259 KB PPT)

Figure S6 CaFT Profiling and Characterization of Brefeldin A (101 KB PPT)

Figure S7 CaFT Profiles of Cytochalasin D (78 KB PPT)

Figure S8 CaFT Profiles of Mycotoxins (177 KB PPT)

Figure S9 CaFT Profiles of 5-FU, 5-FC, Tubercidin, and 6-AU, and Characterization of C. albicans Nucleoside Transporter Nnt1p (72 KB PPT)

Figure S10 Dose-Dependent Inhibition of In Vitro Polymerization of Bovine Tubulin by Nocodazole, ECC85, ECC248, and ECC275 (283 KB PPT)

Table S1 List of Normalized *z*-Scores of the CaFT Experiments (3.9 MB XLS)

Table S2 Summary of Allelic Polymorphism (101 KB DOC)

Text S1 Supplementary Results (91 KB DOC)

## Abbreviations

5-FC: 5-fluorocytosine
5-FU: 5-fluorouracil
CaFT: *C. albicans* fitness test
FAS: fatty acid synthase
HI: haploinsufficiency
IC: inhibitory concentration
MOA: mechanism of action
ORF: open reading frame
ScFT: *S. cerevisiae* fitness test
UPRT: uracil phosphoribosyltransferase
