# Supplementary material for: Genome-Wide Fitness Test and Mechanism-of-Action Studies of Inhibitory Compounds in Candida albicans
Source: PLoS Pathog. 2007 Jun 29;3(6):e92. doi: 10.1371/journal.ppat.0030092 (PMC1904411; doi:10.1371/journal.ppat.0030092)
Supplement: Figure S1 — (222 KB PPT) [file ppat.0030092.sg001.ppt]

## Slide 1
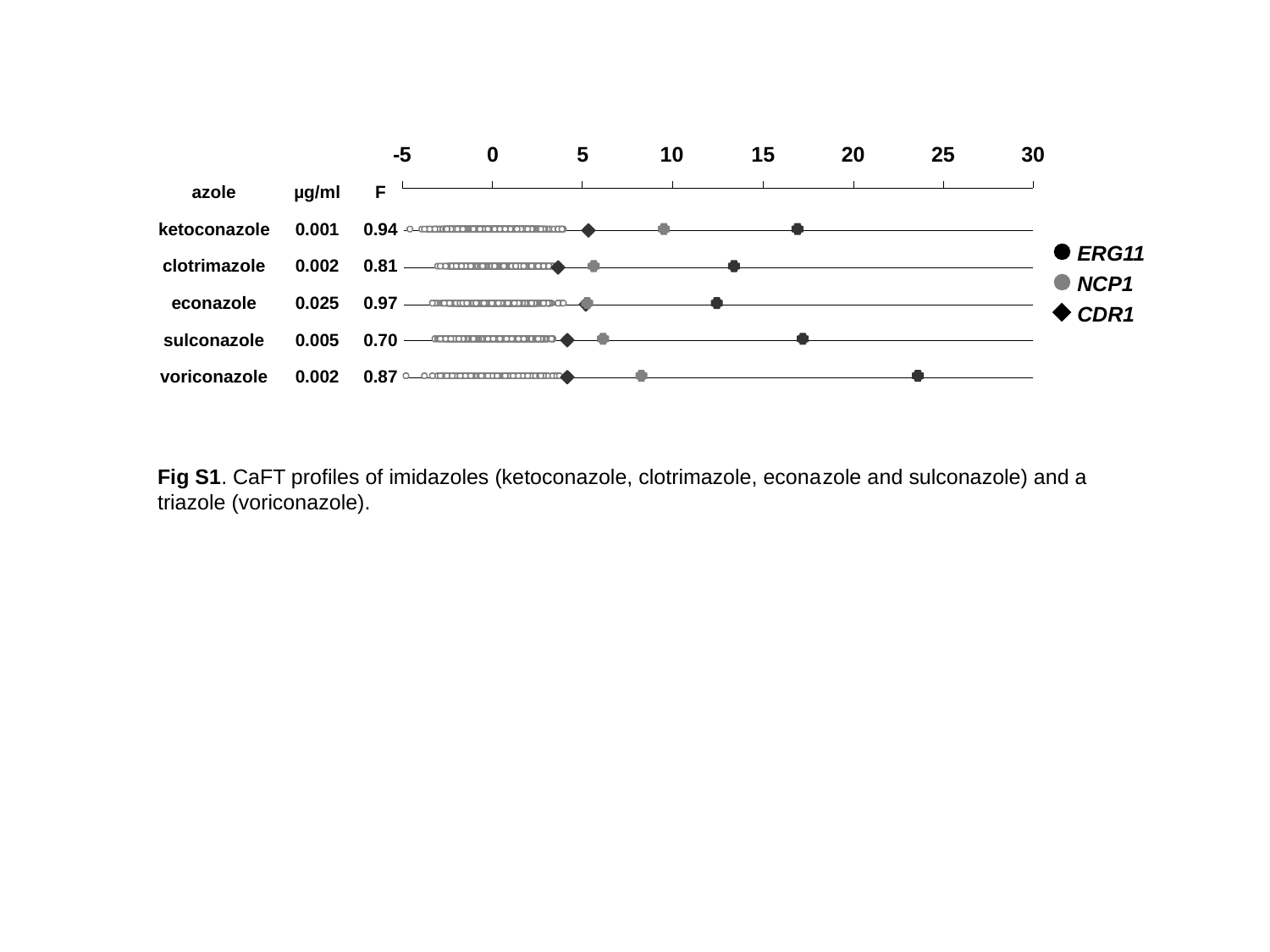

azole	µg/ml	F
	ketoconazole	0.001	0.94
	clotrimazole	0.002	0.81
	econazole	0.025	0.97
	sulconazole	0.005	0.70
	voriconazole	0.002	0.87
ERG11
NCP1
CDR1
Fig S1. CaFT profiles of imidazoles (ketoconazole, clotrimazole, econazole and sulconazole) and a triazole (voriconazole).
