# Supplementary material for: Genome-Wide Fitness Test and Mechanism-of-Action Studies of Inhibitory Compounds in Candida albicans
Source: PLoS Pathog. 2007 Jun 29;3(6):e92. doi: 10.1371/journal.ppat.0030092 (PMC1904411; doi:10.1371/journal.ppat.0030092)
Supplement: Figure S2 — (389 KB PPT) [file ppat.0030092.sg002.ppt]

## Slide 1
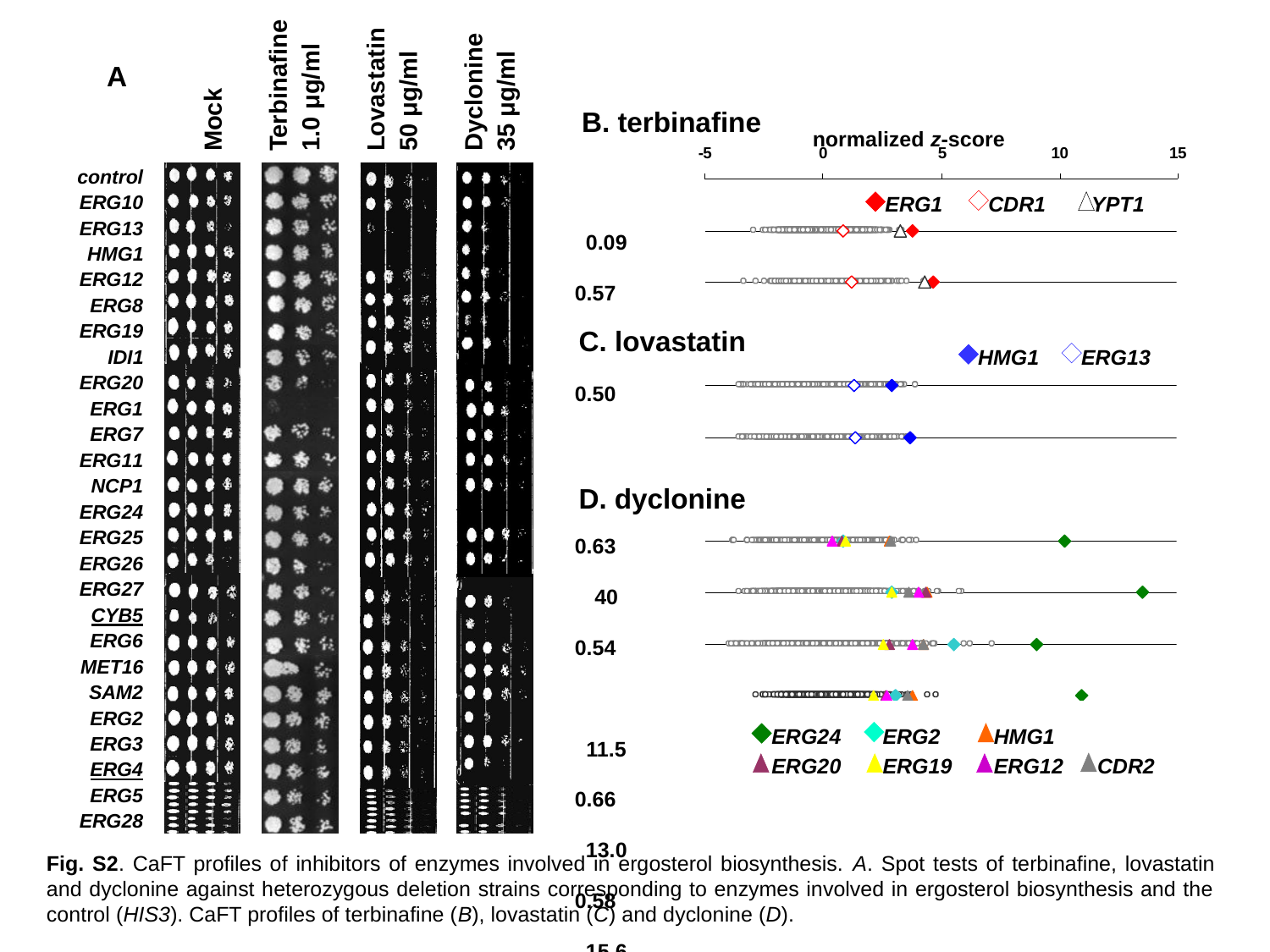

Mock
Terbinafine
1.0 μg/ml
Lovastatin
50 μg/ml
Dyclonine
35 μg/ml
A
B. terbinafine
normalized z-score
ERG1 	CDR1 	YPT1
	0.09	0.57
	0.11	0.50
	30	0.63
	40	0.54
	11.5	0.66
	13.0	0.58
	15.6	0.33
 average
	 μg/ml	F
C. lovastatin
HMG1 	ERG13
D. dyclonine
ERG24	ERG2	HMG1
ERG20	ERG19	ERG12	CDR2
Fig. S2. CaFT profiles of inhibitors of enzymes involved in ergosterol biosynthesis. A. Spot tests of terbinafine, lovastatin and dyclonine against heterozygous deletion strains corresponding to enzymes involved in ergosterol biosynthesis and the control (HIS3). CaFT profiles of terbinafine (B), lovastatin (C) and dyclonine (D).
