# Supplementary material for: Genome-Wide Fitness Test and Mechanism-of-Action Studies of Inhibitory Compounds in Candida albicans
Source: PLoS Pathog. 2007 Jun 29;3(6):e92. doi: 10.1371/journal.ppat.0030092 (PMC1904411; doi:10.1371/journal.ppat.0030092)
Supplement: Figure S3 — (189 KB PPT) [file ppat.0030092.sg003.ppt]

## Slide 1
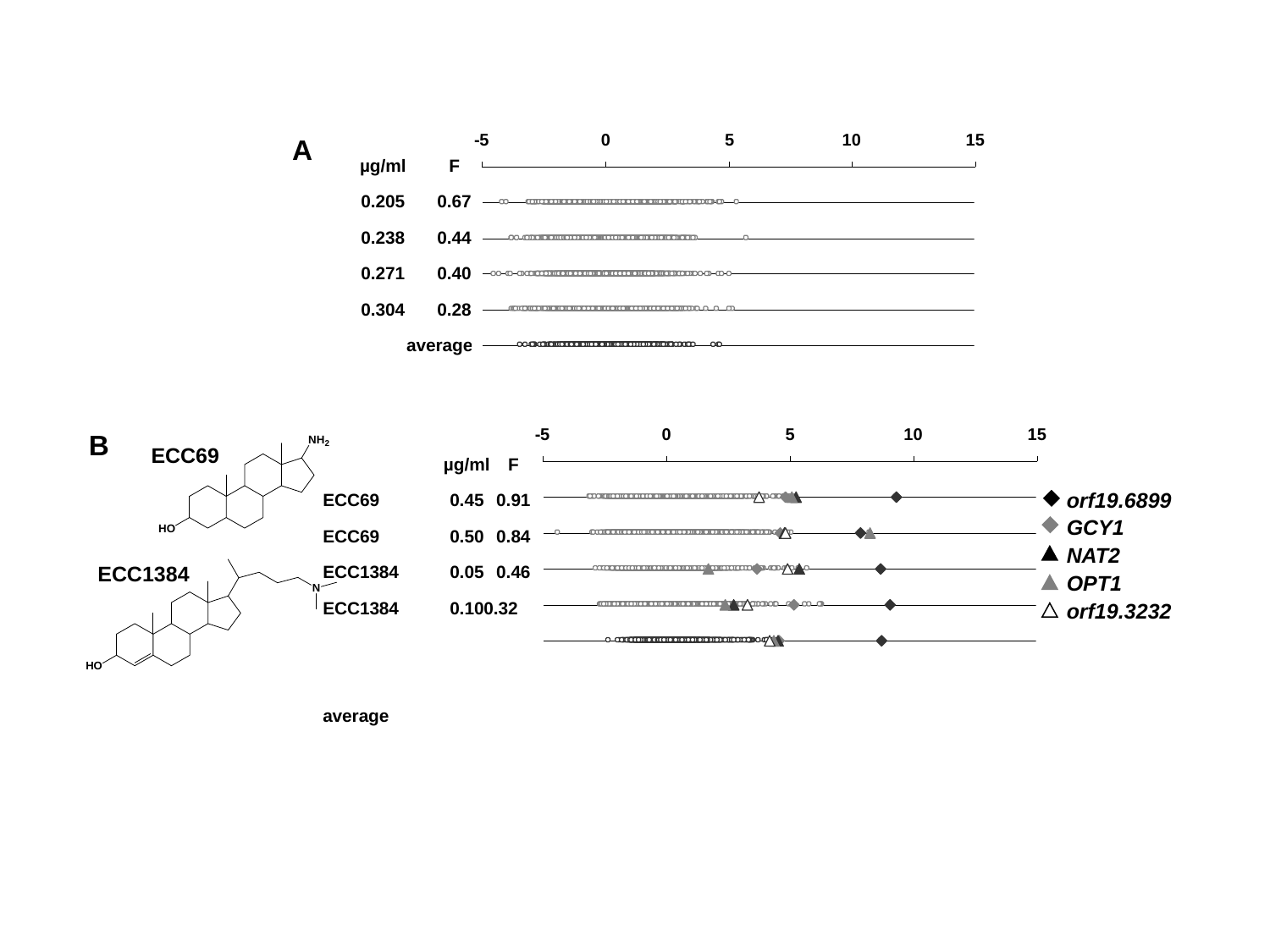

A
	µg/ml	F
	0.205	0.67
	0.238	0.44
	0.271	0.40
	0.304	0.28
	 average
B
	µg/ml	F
ECC69	0.45	0.91
ECC69	0.50	0.84
ECC1384	0.05	0.46
ECC1384	0.10	0.32
		average
ECC69
orf19.6899
GCY1
NAT2
OPT1
orf19.3232
ECC1384

## Slide 2
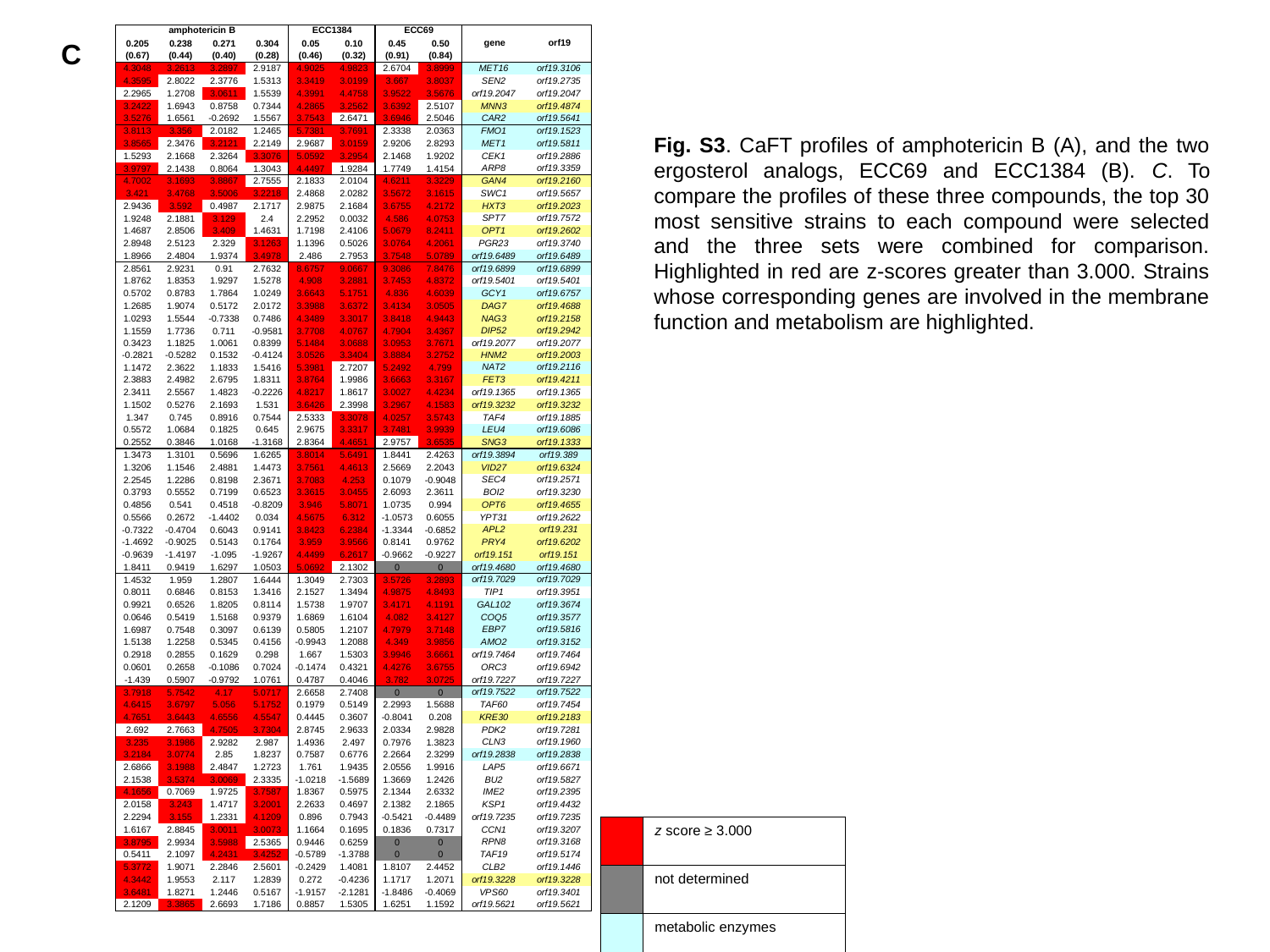

C
Fig. S3. CaFT profiles of amphotericin B (A), and the two ergosterol analogs, ECC69 and ECC1384 (B). C. To compare the profiles of these three compounds, the top 30 most sensitive strains to each compound were selected and the three sets were combined for comparison. Highlighted in red are z-scores greater than 3.000. Strains whose corresponding genes are involved in the membrane function and metabolism are highlighted.
| | z score ≥ 3.000 |
| --- | --- |
| | not determined |
| | metabolic enzymes |
| | membrane related functions |
