# Supplementary material for: Genome-Wide Fitness Test and Mechanism-of-Action Studies of Inhibitory Compounds in Candida albicans
Source: PLoS Pathog. 2007 Jun 29;3(6):e92. doi: 10.1371/journal.ppat.0030092 (PMC1904411; doi:10.1371/journal.ppat.0030092)
Supplement: Figure S4 — (2.4 MB PPT) [file ppat.0030092.sg004.ppt]

## Slide 1
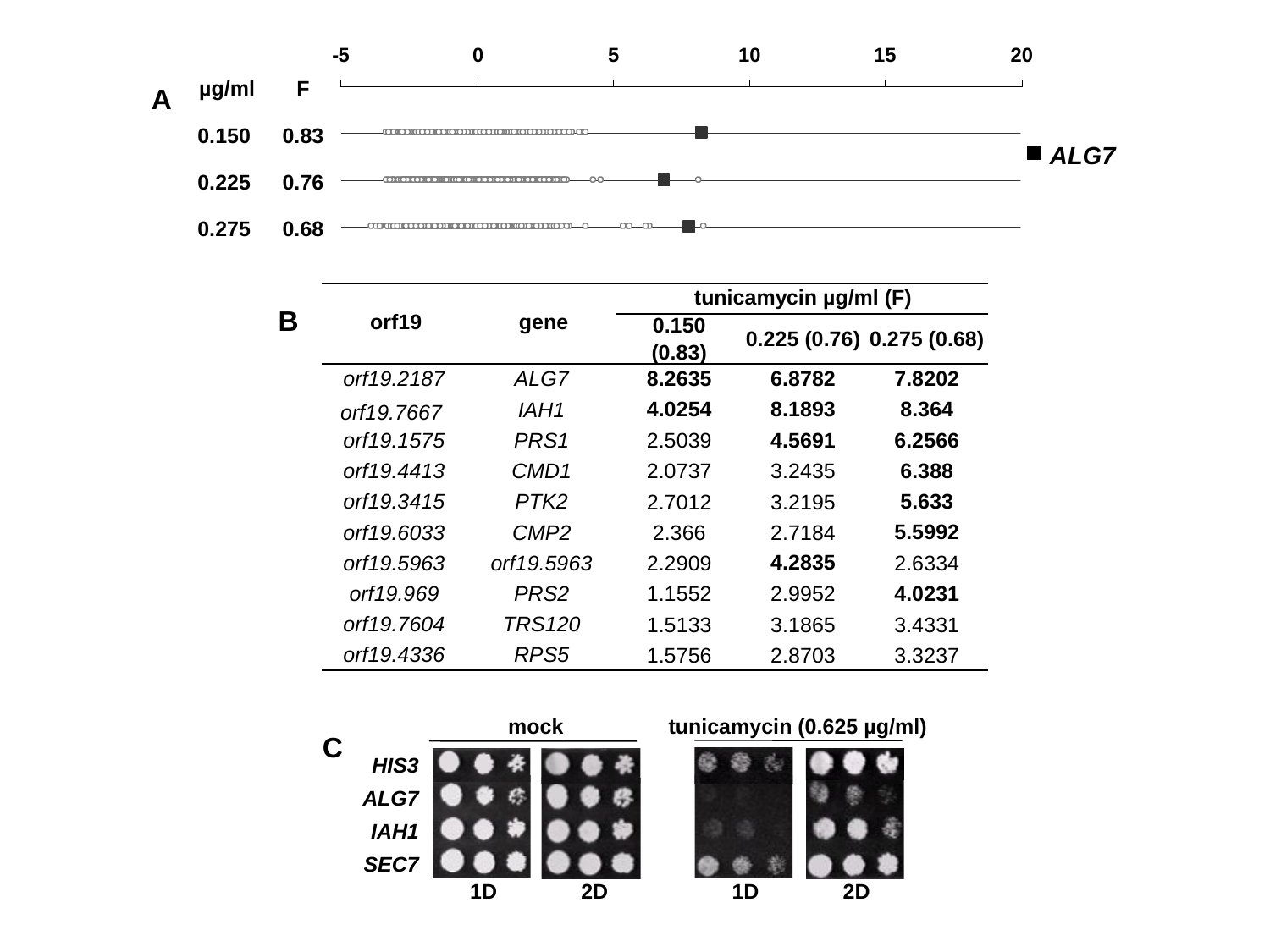

µg/ml	F
	0.150	0.83
	0.225	0.76
	0.275	0.68
A
ALG7
B
	mock	tunicamycin (0.625 µg/ml)
C
HIS3
ALG7
IAH1
SEC7
	1D	2D	1D	2D

## Slide 2
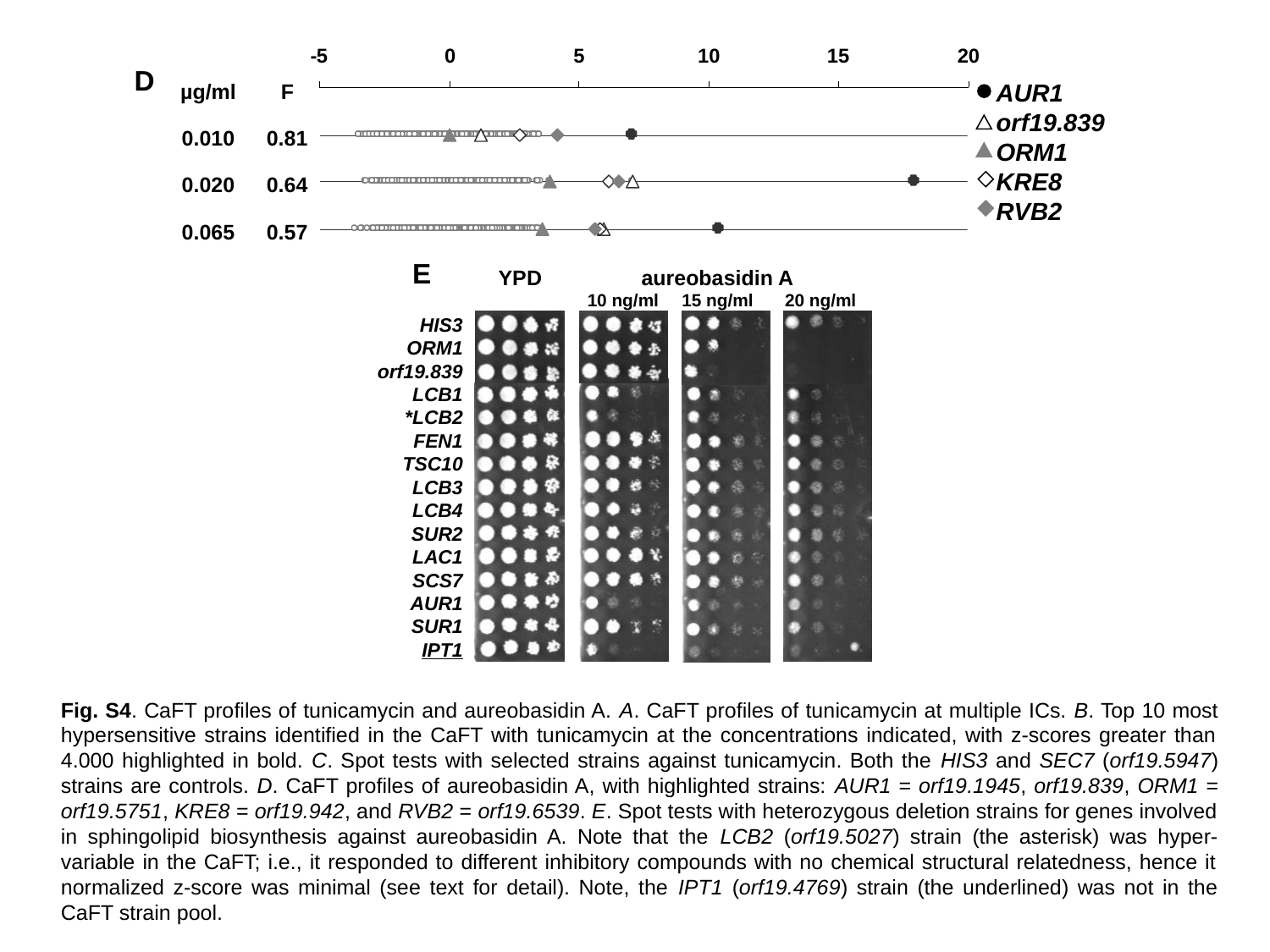

µg/ml	F
	0.010	0.81
	0.020	0.64
	0.065	0.57
D
AUR1
orf19.839
ORM1
KRE8
RVB2
E
	YPD		aureobasidin A
		10 ng/ml	15 ng/ml	20 ng/ml
HIS3
ORM1
orf19.839
LCB1
*LCB2
FEN1
TSC10
LCB3
LCB4
SUR2
LAC1
SCS7
AUR1
SUR1
IPT1
Fig. S4. CaFT profiles of tunicamycin and aureobasidin A. A. CaFT profiles of tunicamycin at multiple ICs. B. Top 10 most hypersensitive strains identified in the CaFT with tunicamycin at the concentrations indicated, with z-scores greater than 4.000 highlighted in bold. C. Spot tests with selected strains against tunicamycin. Both the HIS3 and SEC7 (orf19.5947) strains are controls. D. CaFT profiles of aureobasidin A, with highlighted strains: AUR1 = orf19.1945, orf19.839, ORM1 = orf19.5751, KRE8 = orf19.942, and RVB2 = orf19.6539. E. Spot tests with heterozygous deletion strains for genes involved in sphingolipid biosynthesis against aureobasidin A. Note that the LCB2 (orf19.5027) strain (the asterisk) was hyper-variable in the CaFT; i.e., it responded to different inhibitory compounds with no chemical structural relatedness, hence it normalized z-score was minimal (see text for detail). Note, the IPT1 (orf19.4769) strain (the underlined) was not in the CaFT strain pool.
