# Supplementary material for: Genome-Wide Fitness Test and Mechanism-of-Action Studies of Inhibitory Compounds in Candida albicans
Source: PLoS Pathog. 2007 Jun 29;3(6):e92. doi: 10.1371/journal.ppat.0030092 (PMC1904411; doi:10.1371/journal.ppat.0030092)
Supplement: Figure S5 — (259 KB PPT) [file ppat.0030092.sg005.ppt]

## Slide 1
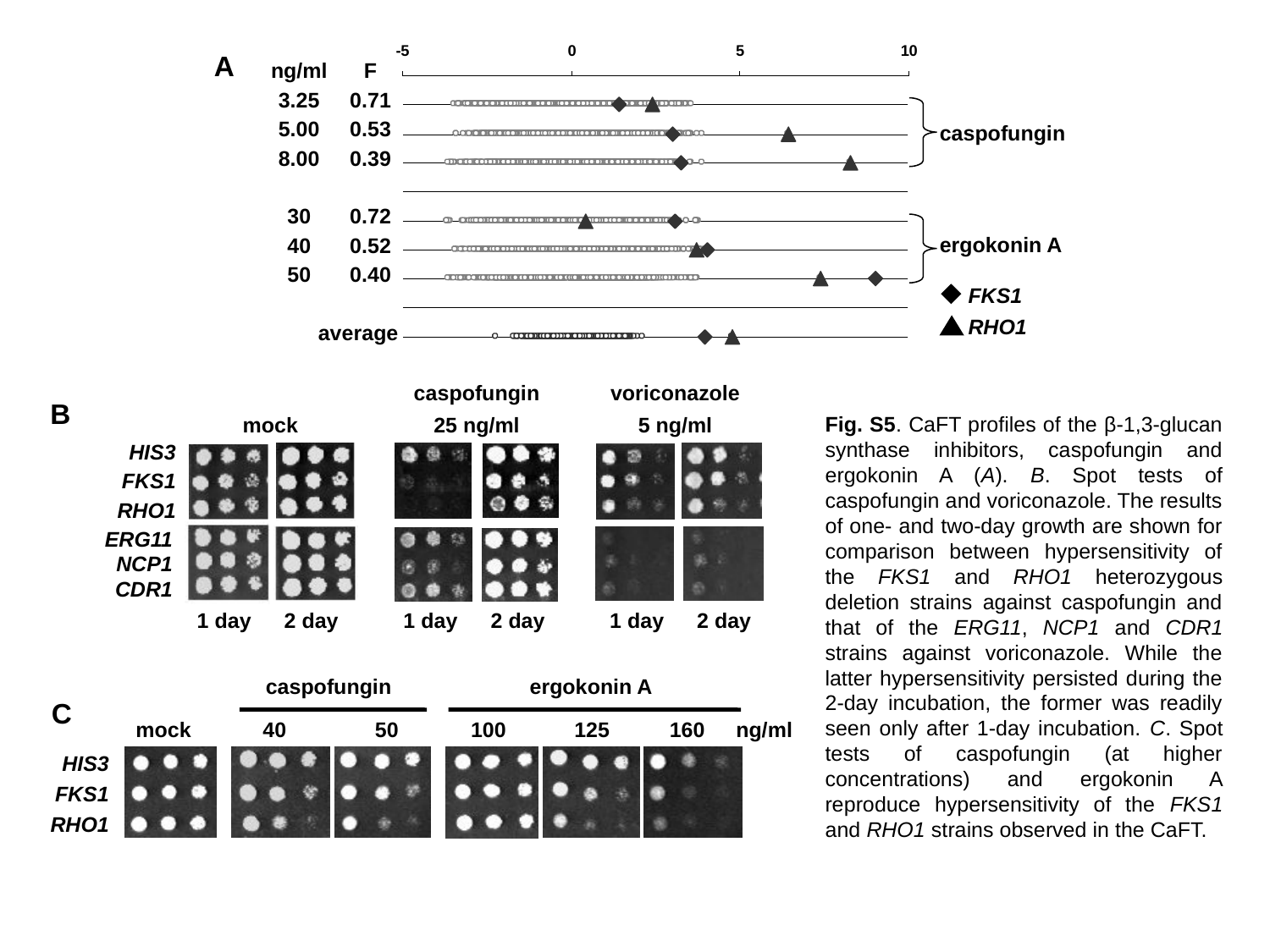

ng/ml	F
	3.25	0.71
	5.00	0.53
	8.00	0.39
	30	0.72
	40	0.52
	50	0.40
	average
A
caspofungin
ergokonin A
FKS1
RHO1
		caspofungin	voriconazole
	mock	25 ng/ml	5 ng/ml
B
HIS3
FKS1
RHO1
ERG11
NCP1
CDR1
	1 day	2 day	1 day	2 day	1 day	2 day
Fig. S5. CaFT profiles of the β-1,3-glucan synthase inhibitors, caspofungin and ergokonin A (A). B. Spot tests of caspofungin and voriconazole. The results of one- and two-day growth are shown for comparison between hypersensitivity of the FKS1 and RHO1 heterozygous deletion strains against caspofungin and that of the ERG11, NCP1 and CDR1 strains against voriconazole. While the latter hypersensitivity persisted during the 2-day incubation, the former was readily seen only after 1-day incubation. C. Spot tests of caspofungin (at higher concentrations) and ergokonin A reproduce hypersensitivity of the FKS1 and RHO1 strains observed in the CaFT.
	caspofungin	ergokonin A
C
	mock	40	50	100	125	160	ng/ml
HIS3
FKS1
RHO1
