# Supplementary material for: Genome-Wide Fitness Test and Mechanism-of-Action Studies of Inhibitory Compounds in Candida albicans
Source: PLoS Pathog. 2007 Jun 29;3(6):e92. doi: 10.1371/journal.ppat.0030092 (PMC1904411; doi:10.1371/journal.ppat.0030092)
Supplement: Figure S6 — (101 KB PPT) [file ppat.0030092.sg006.ppt]

## Slide 1
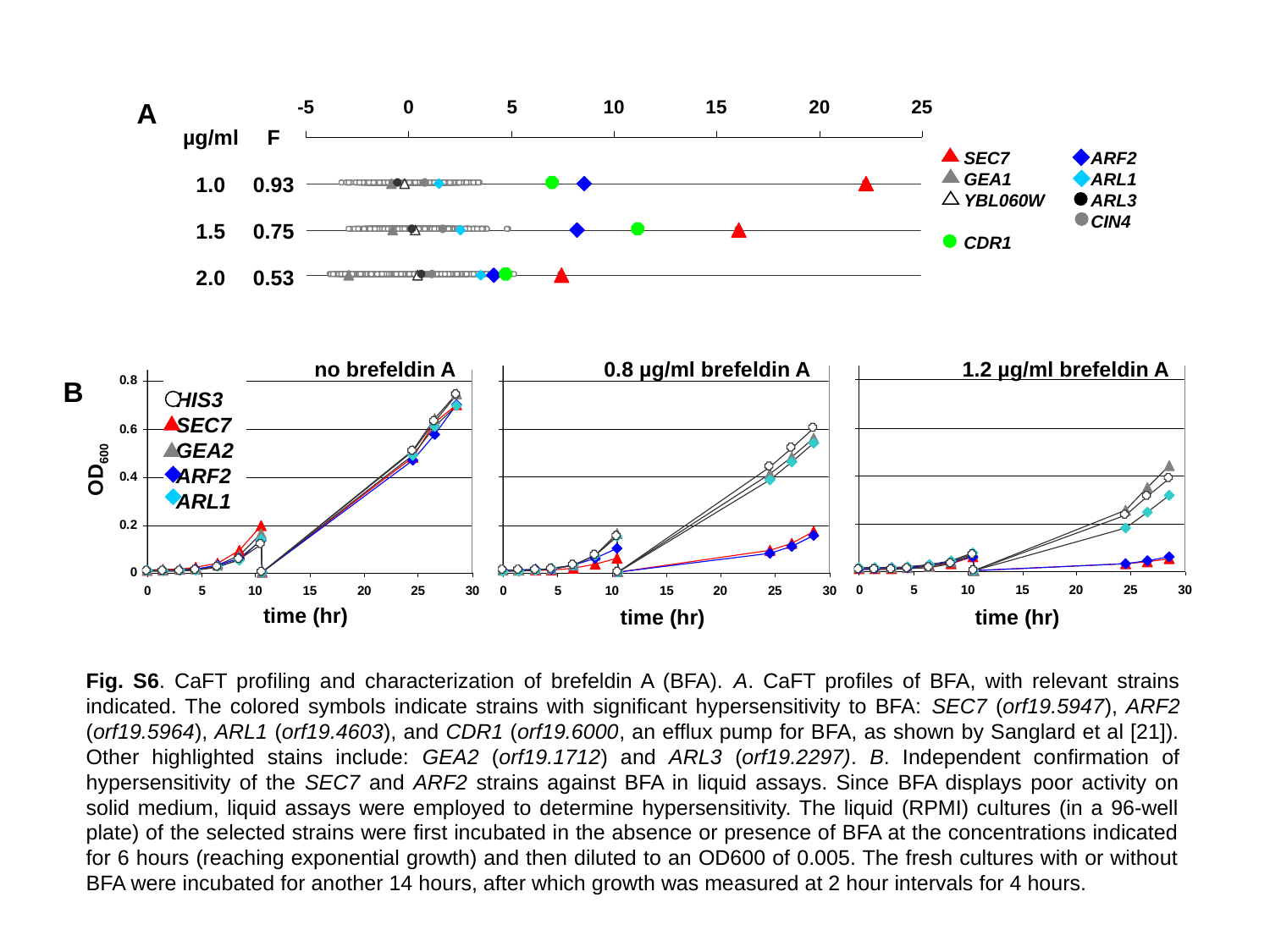

A
	µg/ml	F
	1.0	0.93
	1.5	0.75
	2.0	0.53
SEC7	ARF2
GEA1	ARL1
YBL060W 	ARL3
	CIN4
CDR1
no brefeldin A
0.8 µg/ml brefeldin A
1.2 µg/ml brefeldin A
B
HIS3
SEC7
GEA2
ARF2
ARL1
OD600
time (hr)
time (hr)
time (hr)
Fig. S6. CaFT profiling and characterization of brefeldin A (BFA). A. CaFT profiles of BFA, with relevant strains indicated. The colored symbols indicate strains with significant hypersensitivity to BFA: SEC7 (orf19.5947), ARF2 (orf19.5964), ARL1 (orf19.4603), and CDR1 (orf19.6000, an efflux pump for BFA, as shown by Sanglard et al [21]). Other highlighted stains include: GEA2 (orf19.1712) and ARL3 (orf19.2297). B. Independent confirmation of hypersensitivity of the SEC7 and ARF2 strains against BFA in liquid assays. Since BFA displays poor activity on solid medium, liquid assays were employed to determine hypersensitivity. The liquid (RPMI) cultures (in a 96-well plate) of the selected strains were first incubated in the absence or presence of BFA at the concentrations indicated for 6 hours (reaching exponential growth) and then diluted to an OD600 of 0.005. The fresh cultures with or without BFA were incubated for another 14 hours, after which growth was measured at 2 hour intervals for 4 hours.
