# Supplementary material for: Genome-Wide Fitness Test and Mechanism-of-Action Studies of Inhibitory Compounds in Candida albicans
Source: PLoS Pathog. 2007 Jun 29;3(6):e92. doi: 10.1371/journal.ppat.0030092 (PMC1904411; doi:10.1371/journal.ppat.0030092)
Supplement: Figure S7 — (78 KB PPT) [file ppat.0030092.sg007.ppt]

## Slide 1
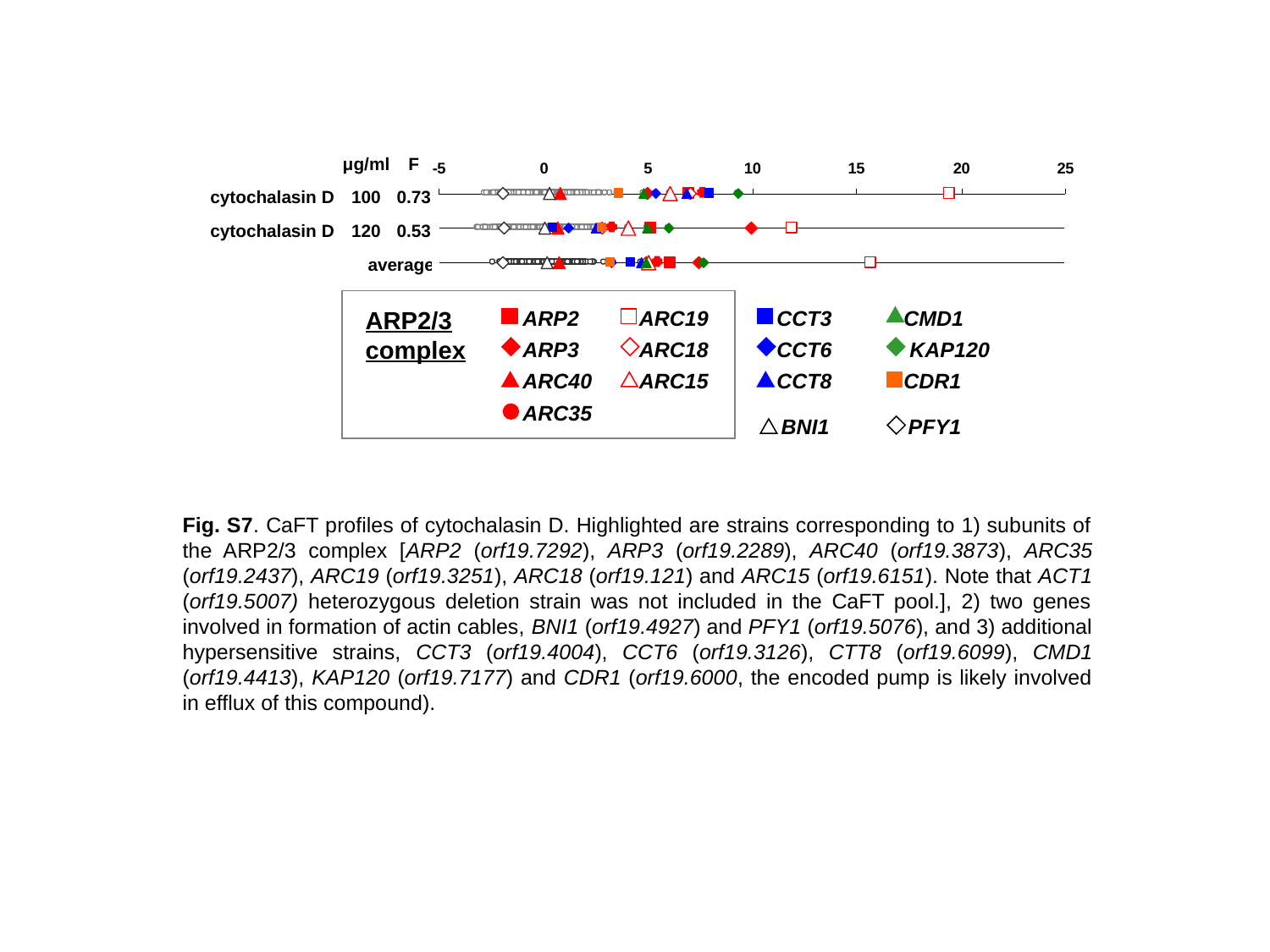

μg/ml	F
	cytochalasin D	100	0.73
	cytochalasin D	120	0.53
average
ARP2	 ARC19 	CCT3	CMD1
ARP3	 ARC18 	CCT6	 KAP120
ARC40	 ARC15 	CCT8	CDR1
ARC35
ARP2/3
complex
BNI1	PFY1
Fig. S7. CaFT profiles of cytochalasin D. Highlighted are strains corresponding to 1) subunits of the ARP2/3 complex [ARP2 (orf19.7292), ARP3 (orf19.2289), ARC40 (orf19.3873), ARC35 (orf19.2437), ARC19 (orf19.3251), ARC18 (orf19.121) and ARC15 (orf19.6151). Note that ACT1 (orf19.5007) heterozygous deletion strain was not included in the CaFT pool.], 2) two genes involved in formation of actin cables, BNI1 (orf19.4927) and PFY1 (orf19.5076), and 3) additional hypersensitive strains, CCT3 (orf19.4004), CCT6 (orf19.3126), CTT8 (orf19.6099), CMD1 (orf19.4413), KAP120 (orf19.7177) and CDR1 (orf19.6000, the encoded pump is likely involved in efflux of this compound).
