# Supplementary material for: Genome-Wide Fitness Test and Mechanism-of-Action Studies of Inhibitory Compounds in Candida albicans
Source: PLoS Pathog. 2007 Jun 29;3(6):e92. doi: 10.1371/journal.ppat.0030092 (PMC1904411; doi:10.1371/journal.ppat.0030092)
Supplement: Figure S8 — (177 KB PPT) [file ppat.0030092.sg008.ppt]

## Slide 1
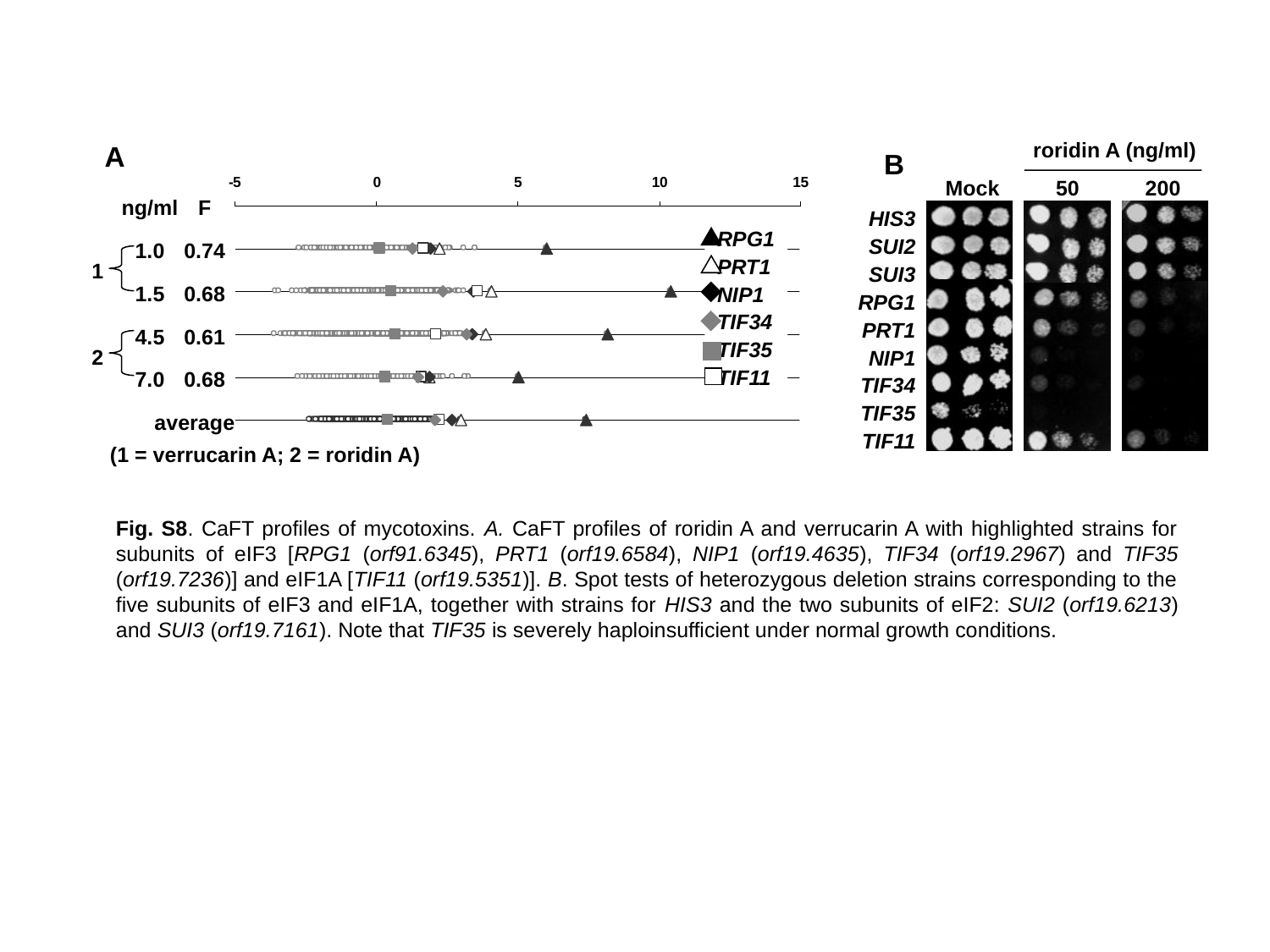

roridin A (ng/ml)
B
	Mock	50	200
HIS3
SUI2
SUI3
RPG1
PRT1
NIP1
TIF34
TIF35
TIF11
A
	ng/ml	F
	1.0	0.74
	1.5	0.68
	4.5	0.61
	7.0	0.68
average
RPG1
PRT1
NIP1
TIF34
TIF35
TIF11
1
2
(1 = verrucarin A; 2 = roridin A)
Fig. S8. CaFT profiles of mycotoxins. A. CaFT profiles of roridin A and verrucarin A with highlighted strains for subunits of eIF3 [RPG1 (orf91.6345), PRT1 (orf19.6584), NIP1 (orf19.4635), TIF34 (orf19.2967) and TIF35 (orf19.7236)] and eIF1A [TIF11 (orf19.5351)]. B. Spot tests of heterozygous deletion strains corresponding to the five subunits of eIF3 and eIF1A, together with strains for HIS3 and the two subunits of eIF2: SUI2 (orf19.6213) and SUI3 (orf19.7161). Note that TIF35 is severely haploinsufficient under normal growth conditions.
