# Supplementary material for: Genome-Wide Fitness Test and Mechanism-of-Action Studies of Inhibitory Compounds in Candida albicans
Source: PLoS Pathog. 2007 Jun 29;3(6):e92. doi: 10.1371/journal.ppat.0030092 (PMC1904411; doi:10.1371/journal.ppat.0030092)
Supplement: Figure S9 — (72 KB PPT) [file ppat.0030092.sg009.ppt]

## Slide 1
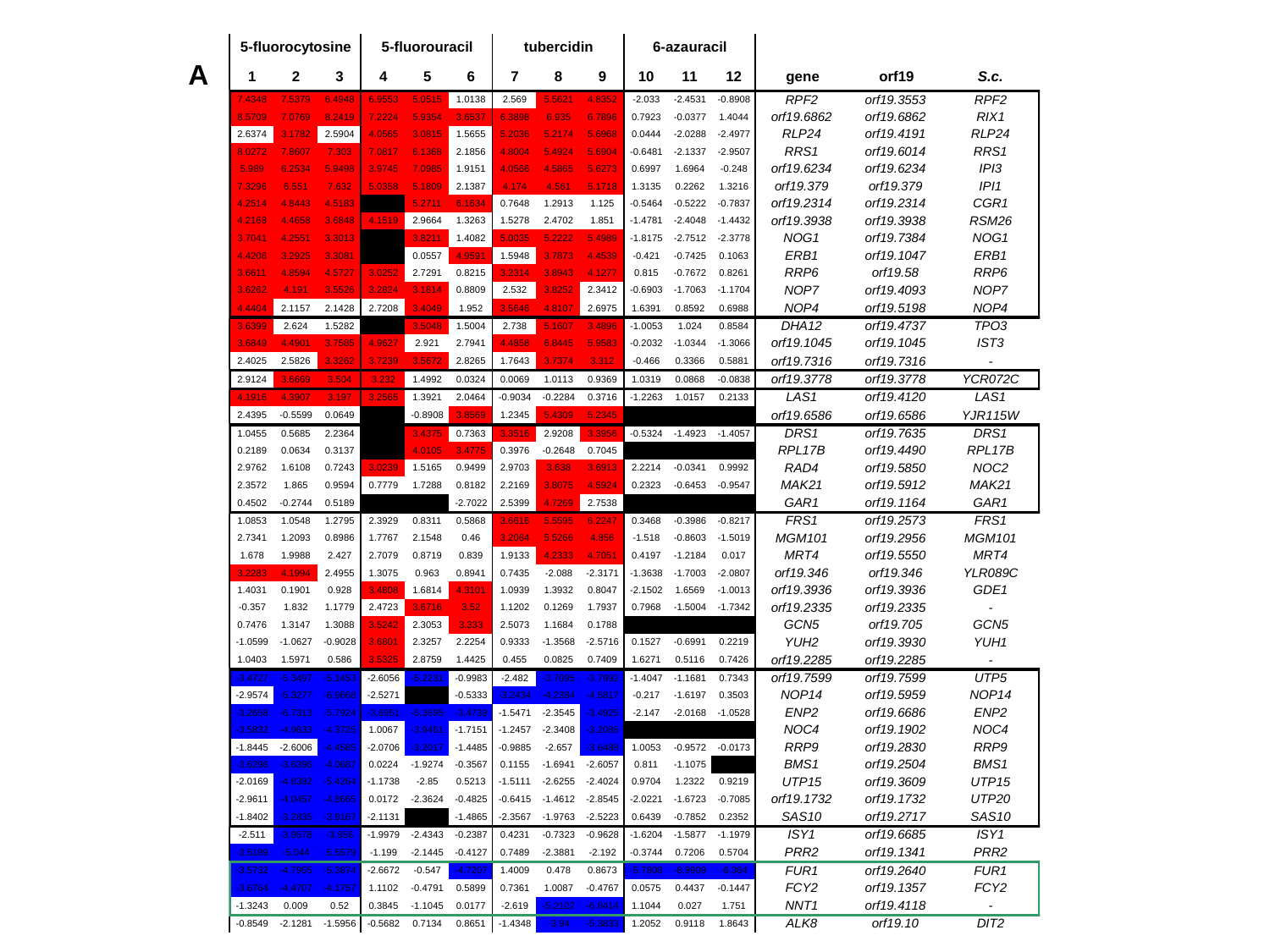

A

## Slide 2
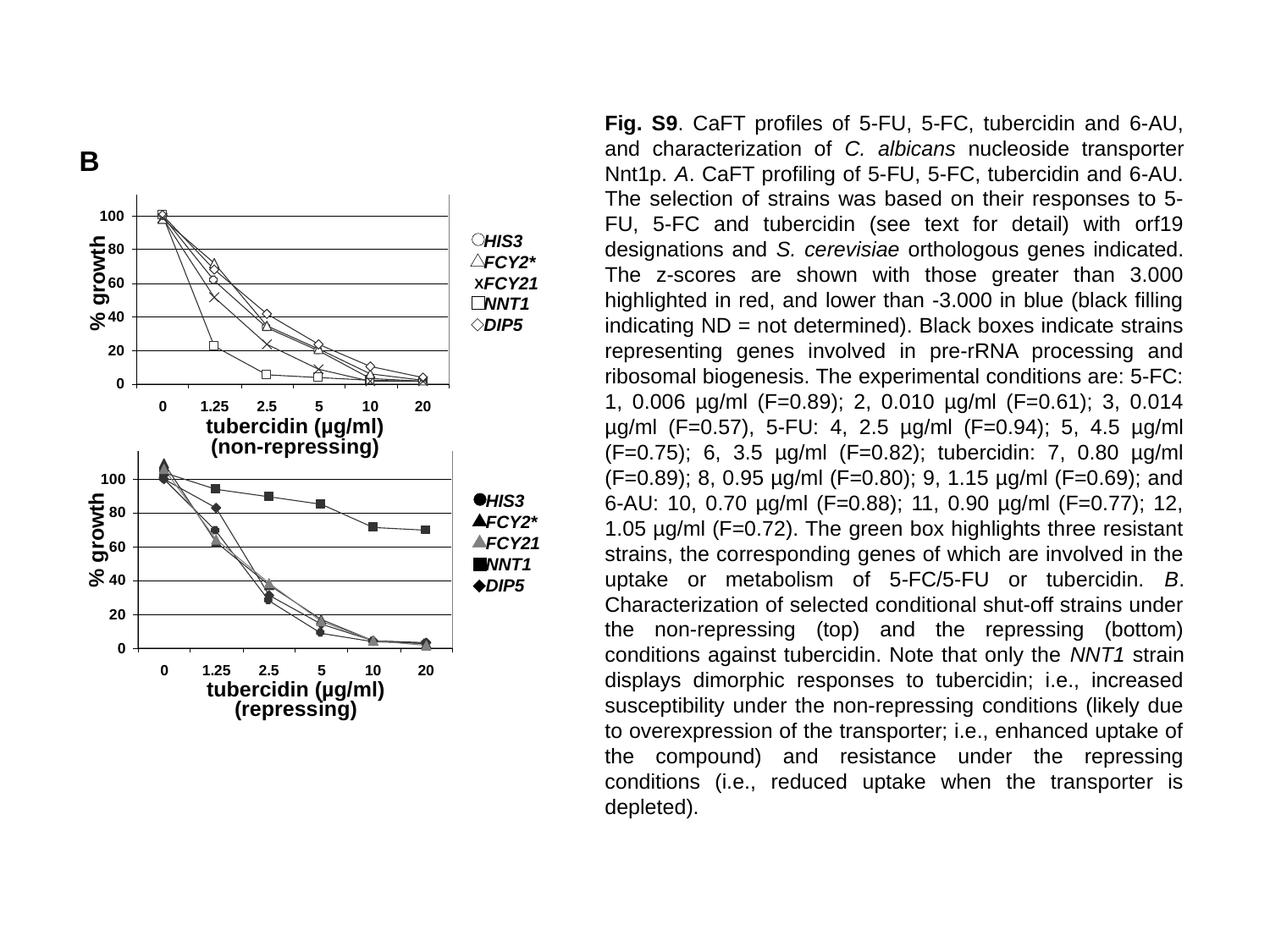

Fig. S9. CaFT profiles of 5-FU, 5-FC, tubercidin and 6-AU, and characterization of C. albicans nucleoside transporter Nnt1p. A. CaFT profiling of 5-FU, 5-FC, tubercidin and 6-AU. The selection of strains was based on their responses to 5-FU, 5-FC and tubercidin (see text for detail) with orf19 designations and S. cerevisiae orthologous genes indicated. The z-scores are shown with those greater than 3.000 highlighted in red, and lower than -3.000 in blue (black filling indicating ND = not determined). Black boxes indicate strains representing genes involved in pre-rRNA processing and ribosomal biogenesis. The experimental conditions are: 5-FC: 1, 0.006 µg/ml (F=0.89); 2, 0.010 µg/ml (F=0.61); 3, 0.014 µg/ml (F=0.57), 5-FU: 4, 2.5 µg/ml (F=0.94); 5, 4.5 µg/ml (F=0.75); 6, 3.5 µg/ml (F=0.82); tubercidin: 7, 0.80 µg/ml (F=0.89); 8, 0.95 µg/ml (F=0.80); 9, 1.15 µg/ml (F=0.69); and 6-AU: 10, 0.70 µg/ml (F=0.88); 11, 0.90 µg/ml (F=0.77); 12, 1.05 µg/ml (F=0.72). The green box highlights three resistant strains, the corresponding genes of which are involved in the uptake or metabolism of 5-FC/5-FU or tubercidin. B. Characterization of selected conditional shut-off strains under the non-repressing (top) and the repressing (bottom) conditions against tubercidin. Note that only the NNT1 strain displays dimorphic responses to tubercidin; i.e., increased susceptibility under the non-repressing conditions (likely due to overexpression of the transporter; i.e., enhanced uptake of the compound) and resistance under the repressing conditions (i.e., reduced uptake when the transporter is depleted).
B
HIS3
FCY2*
FCY21
NNT1
DIP5
X
% growth
tubercidin (µg/ml)
(non-repressing)
HIS3
FCY2*
FCY21
NNT1
DIP5
% growth
tubercidin (µg/ml)
(repressing)
