# Supplementary material for: Genome-Wide Fitness Test and Mechanism-of-Action Studies of Inhibitory Compounds in Candida albicans
Source: PLoS Pathog. 2007 Jun 29;3(6):e92. doi: 10.1371/journal.ppat.0030092 (PMC1904411; doi:10.1371/journal.ppat.0030092)
Supplement: Figure S10 — (283 KB PPT) [file ppat.0030092.sg010.ppt]

## Slide 1
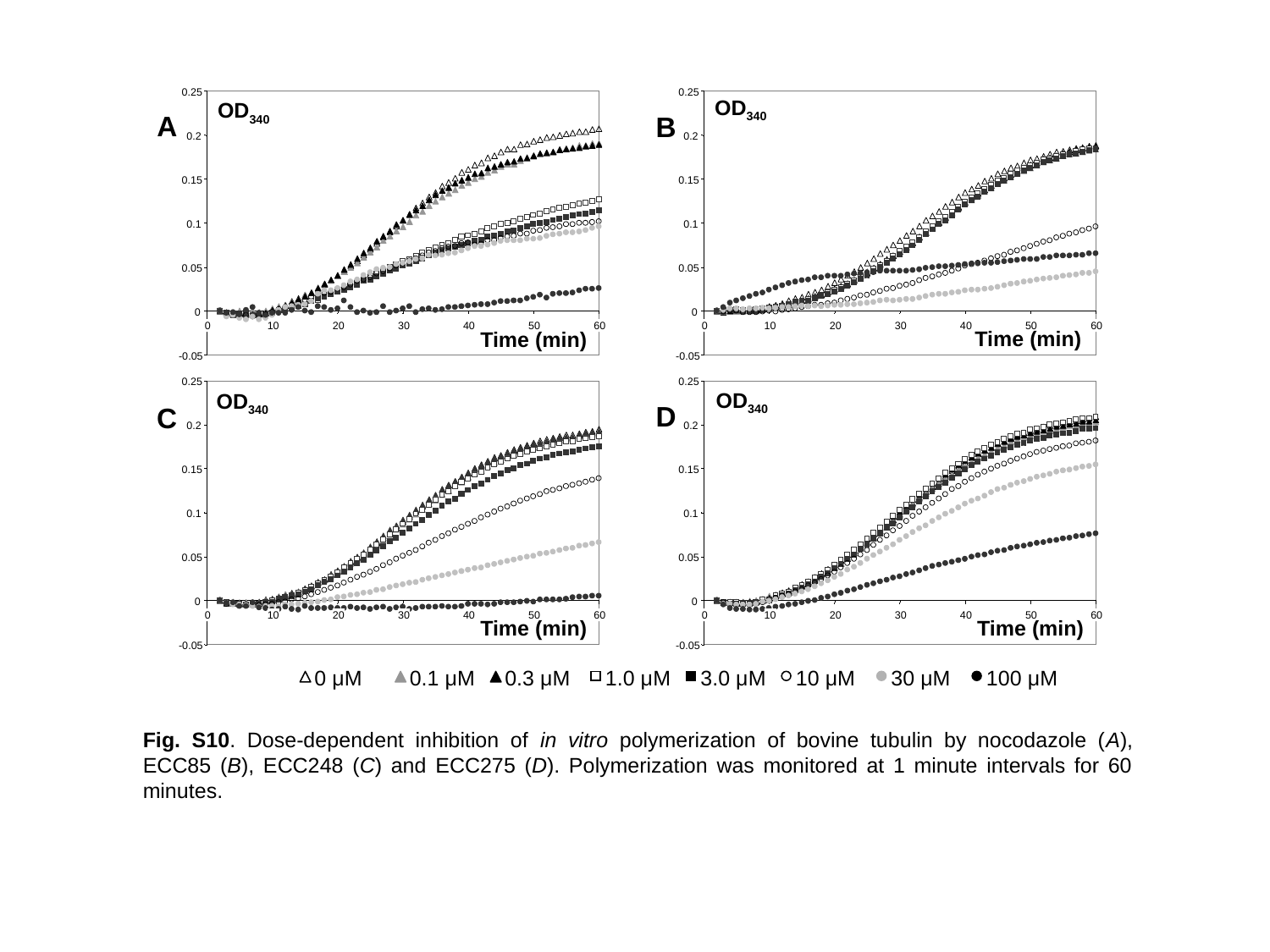

0.25
OD340
0.2
0.15
0.1
0.05
0
0
10
20
30
40
50
60
Time (min)
-0.05
0.25
OD340
0.2
0.15
0.1
0.05
0
Time (min)
0
10
20
30
40
50
60
-0.05
A
B
0.25
OD340
0.2
0.15
0.1
0.05
0
Time (min)
0
10
20
30
40
50
60
-0.05
0.25
OD340
0.2
0.15
0.1
0.05
0
Time (min)
0
10
20
30
40
50
60
-0.05
D
C
0 μM
0.1 μM
0.3 μM
1.0 μM
3.0 μM
10 μM
30 μM
100 μM
Fig. S10. Dose-dependent inhibition of in vitro polymerization of bovine tubulin by nocodazole (A), ECC85 (B), ECC248 (C) and ECC275 (D). Polymerization was monitored at 1 minute intervals for 60 minutes.
