# Supplementary material for: Genome-Wide Fitness Test and Mechanism-of-Action Studies of Inhibitory Compounds in Candida albicans
Source: PLoS Pathog. 2007 Jun 29;3(6):e92. doi: 10.1371/journal.ppat.0030092 (PMC1904411; doi:10.1371/journal.ppat.0030092)
Supplement: Table S2 — (101 KB DOC) [file ppat.0030092.st002.doc]

**Table S2**. Summary of allelic polymorphism.

| **ORF19 Designation** | **Gene Name** | **Allelic Identity** |
| --- | --- | --- |
| Group 1. Strains that are hypersensitive to compounds | | |
| orf19.922 | *ERG11* | 0.99 |
| orf19.2672 | *NCP1* | 1.00 |
| orf19.6000 | *PDR5* | 1.00 |
| orf19.5839 | *PDR16* | 1.00 |
| orf19.1598 | *ERG24* | 1.00 |
| orf19.3106 | *MET16* | 1.00 |
| orf19.2942 | *DIP5* | 1.00 |
| orf19.2116 | *NAT2* | 0.99 |
| orf19.2077 | *ARG81* | 1.00 |
| orf19.2160 | *TPO3* | 1.00 |
| orf19.1523 |  | 1.00 |
| orf19.698 | *YIL090w* | 1.00 |
| orf19.2003 | *HNM1* | 0.99 |
| orf19.7522 |  | 1.00 |
| orf19.7454 | *TAF6* | 1.00 |
| orf19.2183 | *YER036c* | 1.00 |
| orf19.7281 |  | 1.00 |
| orf19.5657 | *SWI1* | 1.00 |
| orf19.7197 | *NOC3* | 1.00 |
| orf19.2187 | *ALG7* | 1.00 |
| orf19.5947 | *SEC7* | 1.00 |
| orf19.5964 | *ARF2* | 1.00 |
| orf19.3951 | *YIP1* | 1.00 |
| orf19.2629 | *USO1* | 1.00 |
| orf19.6642 | *YIF1* | 1.00 |
| orf19.2571 | *SEC4* | 1.00 |
| orf19.1191 | *HRD3* | 1.00 |
| orf19.3898 | *TLG1* | 1.00 |
| orf19.941 | *SEC14* | 1.00 |
| orf19.4594 | *CLC1* | 1.00 |
| orf19.1251 | *BRN1* | 1.00 |
| orf19.3247 | *YJL207c* | 1.00 |
| orf19.3699 | *TEP1* | 1.00 |
| orf19.7308 | *TUB1* | 1.00 |
| orf19.2678 | *BUB1* | 1.00 |
| orf19.3356 | *ESP1* | 1.00 |
| orf19.1945 | *AUR1* | 1.00 |
| orf19.379 | *IPI1* | 1.00 |
| orf19.6234 | *IPI3* | 0.98 |
| orf19.2314 | *CGR1* | 1.00 |
| orf19.6862 | *RIX1* | 1.00 |
| orf19.4093 | *NOP7* | 1.00 |
| orf19.2917 | *NUG1* | 1.00 |
| orf19.3553 | *RPF2* | 1.00 |
| orf19.6014 | *RRS1* | 0.99 |
| orf19.58 | *RRP6* | 0.99 |
| orf19.3938 | *RSM26* | 1.00 |
| orf19.4492 | *EBP2* | 1.00 |
| orf19.1047 | *ERB1* | 1.00 |
| orf19.5850 | *NOC2* | 1.00 |
| orf19.7384 | *NOG1* | 1.00 |
| orf19.2573 | *FRS1* | 1.00 |
| orf19.4089 | *SGT1* | 1.00 |
| orf19.5531 | *CDC37* | 0.99 |
| orf19.6052 | *CNS1* | 1.00 |
| orf19.6345 | *RPG1* | 0.99 |
| orf19.4635 | *NIP1* | 1.00 |
| orf19.5351 | *TIF11* | 1.00 |
| orf19.3251 | *ARC19* | 1.00 |
| orf19.121 | *ARC18* | 1.00 |
| orf19.7292 | *ARP2* | 1.00 |
| orf19.2289 | *ARP3* | 1.00 |
| orf19.2437 | *ARC35* | 1.00 |
| orf19.6151 | *ARC15* | 1.00 |
| orf19.6099 | *CCT8* | 1.00 |
| orf19.4004 | *CCT3* | 1.00 |
| orf19.3126 | *CCT6* | 1.00 |
| orf19.2929 | *FKS1* | 1.00 |
| orf19.2843 | *RHO1* | 1.00 |
| Group 2. Strains that are not hypersensitive to compounds | | |
| orf19.4603 | *ARL1* | 1.00 |
| orf19.5949 | *FAS2* | 1.00 |
| orf19.5604 | *MDR1* | 1.00 |
| orf19.6034 | *TUB2* | 1.00 |
| orf19.4927 | *BNI1* | 1.00 |
| orf19.5076 | *PFY1* | 1.00 |
| orf19.3873 | *ARC40* | 0.99 |
| orf19.7236 | *TIF35* | 1.00 |
| orf19.6515 | *HSC82* | 1.00 |
| orf19.7654 | *CPR6* | 1.00 |
| orf19.7602 | *AHA1* | 1.00 |
| orf19.1040 | *MAD2* | 0.98 |
| orf19.7312 | *ERG13* | 1.00 |
| orf19.1031 | *HMG1* | 1.00 |
| orf19.6026 | *ERG2* | 1.00 |
