# Supplementary material for: Genome-Wide Fitness Test and Mechanism-of-Action Studies of Inhibitory Compounds in Candida albicans
Source: PLoS Pathog. 2007 Jun 29;3(6):e92. doi: 10.1371/journal.ppat.0030092 (PMC1904411; doi:10.1371/journal.ppat.0030092)
Supplement: Text S1 — (91 KB DOC) [file ppat.0030092.sd001.doc]

**Supplementary Results**

The normalized z-scores of all the strains determined in all the experiments described in this study are provided in Table S1. The experimental data are organized according to the order of their appearance in the text, with drug concentrations, F-values and the corresponding figures indicated.

In Table S2, we list genes described in the CaFT profiles, in the text and supplementary results, and their allelic polymorphisms. They are divided into two groups according to the corresponding heterozygous deletion strains: 1) those whose hypersensitivity/resistance was observed in the CaFT experiments; and 2) those whose functions are related to the compounds tested and/or functionally connected to genes in the first group, but whose hypersensitivity/resistance was not observed in the CaFT experiments. Similar levels of allelic polymorphism are observed in both groups, in terms of frequency (8/69 vs. 2/15) and allelic identity, suggesting that the lack of response of the heterozygous deletion strains from the second group is not likely due to allelic difference.

Additional CaFT profiles are provided as follows:

**CaFT Profiling of Inhibitors of Enzymes in Ergosterol Biosynthesis**

In addition to fluconazole (a triazole), a number of inhibitors of Erg11p were tested, including imidazoles (ketoconazole, clotrimazole, econzaole and sulconazole) and another triazole (voriconazole), at different ICs (Fig. S1, and data not shown). The specific hypersensitivity of three heterozygotes most relevant to the MOA of these azoles, *ERG11*, *NCP1*, and *CDR1*, was deviated significantly from the population.

The antifungal terbinafine inhibits squalene monooxygenase (squalene epoxidase, *ERG1*, *orf19.406*). In the spot test, the *ERG1* strain displayed significant and specific hypersensitivity over a range of concentrations (1.0, 2.0 and 3.0 μg/ml, Fig. S2A, and data not shown), that was readily confirmed in the CaFT (Fig. S2B). Although a homozygous deletion of *CDR1* confers hypersensitivity to terbinafine [21], the corresponding heterozygote displayed no significant hypersensitivity at the ICs tested in the CaFT; nor did *ScPDR5* (ortholog of *CDR1*) in the ScFT [13]. In the spot tests, the *CDR1* heterozygous deletion strain showed hypersensitivity to terbinafine at concentrations only > 2.0 μg/ml, while that of the homozygous deletion strain was readily observed (data not shown). The CaFT also revealed another hypersensitive strain, *YPT1* (*orf19.3052*, encoding a GTP-binding protein involved in vesicle transport from ER to Golgi). However, its biological relevance is not clear.

Lovastatin is a competitive inhibitor of 3-hydroxy-3-methyl-glutaryl (HMG)-CoA reductase, an enzyme involved in the early step of cholesterol/ergosterol biosynthesis. In *S. cerevisiae*, there are two encoding genes, *ScHMG1* and *ScHMG2*, the former of which contributes the majority (>80%) of the enzymatic activity. Consequently, the heterozygous deletion strain for *ScHMG1* (but not *ScHMG2*) showed specific hypersensitivity to lovastatin in the ScFT [11,13], as did *ScERG13* [11]. *C. albicans* encodes a single HMG-CoA reductase, *HMG1* (*orf19.1031*). Both *HMG1* and *ERG13* (*orf19.7312*) heterozygotes are hypersensitive to lovastatin by spot tests at ≥40 μg/ml lovastatin (Fig. S2A, and data not shown). In the CaFT the hypersensitivity of *HMG1* was only marginally detected while *ERG13* was not (Fig. S2C). In these two experiments, the F value was determined only after 10 hours of growth, whereas after 15 hours no inhibition was observed for lovastatin under the same or higher (as high as 2 mg/ml) concentrations (data not shown). There is no evidence that the performance of the barcodes of the *HMG1* or *ERG13* strain is unsatisfactory as determined by DNA microarrays. We concluded that the fail to detect significant hypersensitivity of either strain is likely due to lack of potency of lovastatin under the assay condition; i.e., in liquid medium determined (by OD) after 15 hours of growth. The discrepancy between results obtained in liquid assay and spot test (solid medium) is rare for the compounds tested in this study. But it is not unprecedented. Brefeldin A is potent only in the liquid media, but significantly less so on solid media (Fig. S6, and data not shown).

The topical antiseptic dyclonine exerts its effect by decreasing the sodium ion permeability of membranes. However, in the ScFT, *ScERG2* was identified as the most hypersensitive in one study [13], and *ScERG24* in another [11]. In spot tests, dyclonine-induced HI varied depending the concentration; i.e., at 30 μg/ml, only the *ERG24* (*orf19.1598*) strain displayed specific hypersensitivity, and at ≥ 35 μg/ml, an increased number of strains correspond to genes in the ergosterol biosynthesis displayed hypersensitivity (Fig. S2A, and data not shown). Most of these strains were identified as hypersensitive in the CaFT with dyclonine in a concentration dependent manner (Fig. S2D). The *ERG24* heterozygote showed the most significant hypersensitivity across all concentrations tested, and that of *ERG2* was more clearly identified at the highest concentration tested (Fig. S2D). Modest hypersensitivity of the *CDR2* heterozygote was also identified (Fig. S2D), suggesting a potential role of the corresponding protein in the efflux of dyclonine. The effect of dyclonine on ergosterol biosynthesis is currently under investigation.

Unlike the inhibitors of specific enzymes involved in ergosterol biosynthesis, the polyene antifungal drug amphotericin B binds preferentially to ergosterol in the plasma membrane of fungi, forming aqueous pores that result in lethal changes in permeability [60]. CaFT profiles did not indicate a clear protein-target based MOA (Fig. S3A, C, see below). Similarly, the two analogs of ergosterol with antifungal activities, ECC69 and ECC1384, produced CaFT profiles containing strains whose corresponding genes are biologically complex (Fig. S3B, C). When analyzed together, CaFT profiles of the two ergosterol analogs are more related to each other than to amphotericin B. While all three compounds shared profiles to a limited extent, each elicited specific hypersensitive strains in its profile (Fig. S3C). However, their biological relevance is not entirely clear. For example, the three most hypersensitive strains of amphotericin B, *orf19.7522*, *TAF60* (*orf19.7454*) and *KRE30* (*orf19.2183*), correspond to a metabolic enzyme, a transcription factor, and an ABC transport, respectively. Although there is no obvious overlap between the profiles of amphotericin B tested in the ScFT [13] and the CaFT, the former profile also contained a number of hypersensitive strains with no clear biological connection. MOAs of these three compounds are likely different in detail, however, their CaFT profiles are highly enriched in genes involved in membrane related functions and metabolic enzymes (Fig. S3C), a clear indication that, as in the case of amphotericin B, the plasma membrane is grossly affected by the two analogs of ergosterol, and, as a consequence, the metabolism is disturbed to the extent that heterozygosity of genes involved in these two aspects of cellular processes results in a higher propensity of reduced fitness.

**CaFT Profiling of Inhibitors of Other Enzymes**

Tunicamycin inhibits the activity of the UDP-N-acetyl-glucosamine-1-P transferase (encoded by *ALG7*) required for asparagine-linked glycosylation of proteins [61]. In the CaFT, the hypersensitivity of the *ALG7* heterozygote against tunicamycin was significantly differentiated from the population (Fig. S4A). However, this compound also elicited additional hypersensitive strains, one of which, *IAH1*, was specific, while others were dose-dependent (Fig. S4B). Spot tests confirmed the hypersensitivity of *ALG7*. However that of *IAH1* was most prominent after 1 day growth in the spot tests (Fig. S4C, and data not shown). It is interesting to note that genes for calmodulin (*CMD1*), calcineurin (*CMP2*) and phosphoribosylpyrophosphate synthetase (*PRS1* and *PRS2*) are clearly haploinsufficient against high concentrations of tunicamycin. In the ScFT with tunicamycin, *ScALG7* was identified as one of the most significant haploinsufficient genes, including *ScPSR2* [13]. It remains to be determined if these additional hypersensitive strains represent a potential connection between protein glycosylation and other processes or the secondary off-target activity of this compound. In *C. albicans*, it has been shown that the calcineurin signaling is involved in tolerance of tunicamycin [62].

Aureobasidin A is a fungicidal agent with a wide spectrum amongst fungal pathogens. It specifically inhibits the phosphatidylinositol:ceramide phosphoinositol transferase encoded by *AUR1* [63]. CaFT profiling of aureobasidin A identified the *AUR1* heterozygote as the most significant and specific hypersensitive in a range of ICs (Fig. S4D), as confirmed by spot test (Fig. S4E). Several other strains also displayed hypersensitivity (in a dose-dependent manner), most notably, *ORM1* and *YLR143w*. Although neither gene is reported to carry out a functional role in sphingolipid biosynthesis, this remains a possibility. Alternatively, *ORM1* and/or *YLR143w* may reflect secondary target(s) of aureobasidin A that contribute(s) to its known toxicity, given that both proteins share strong homology to human orthologs. Although the hypersensitivity of the *LCB2* strain was noted in the spot tests (Fig. S4E), it was not observed in the CaFT (Fig. S4D and Table S1). The *LCB2* strain has two functional barcodes, but their normalized z scores were minimal. This is due to the general hypersensitivity of the *LCB2* strain to various inhibitory compounds tested (data not shown). The high standard deviation computed for the average growth behavior of this strain (i.e., strain/barcode-specific error model) ostensibly compresses the computed normalized *z*-score and thereby its statistical significance.

**CaFT Profiling of Inhibitors of Enzyme Complexes**

Caspofungin inhibits the biosynthesis of β-1,3-glucan, an essential component of the fungal cell-wall [64]. In *C. albicans*, there are three genes that encode the β-1,3-glucan synthase (GS), *FKS1*/*GSC1*/*GSL2* (*orf19.2929*), *GSL1* (*orf19.2495*) and *GSC2-1* (*orf19.3269*). It has been shown that point mutations in *FKS1* confer resistance to caspofungin [65] and, that activation of Fks1p requires the regulatory subunit Rho1p, a multifunctional GTP-binding protein [66]. Both *FKS1* and *RHO1* (*orf19.2843*) are essential for viability [6] and the corresponding heterozygous deletion strains are present in the CaFT. When tested, caspofungin proved technically challenging due to its high potency and exceedingly steep IC curve. Unexpectedly, the strain corresponding to the catalytic subunit, *FKS1*, displayed no differentiated hypersensitivity to caspofungin in the range of IC achieved in the CaFT, while that of the regulatory subunit, *RHO1*, was dose-dependent (Fig. S5A). In spot tests, a time-restricted hypersensitivity was observed for both strains at low concentrations (Fig. S5B and data not shown) while the *RHO1* strain was discernibly hypersensitive at higher concentrations, even after 2 day incubation (Fig. S5C). [The *IAH1*/tunicamycin (Fig. S4) and *FKS1*/caspofungin (Fig. S5) pairs illustrate the subtle difference in the growth conditions used in the CaFT and the spot test.] To overcome technical issues related to testing caspofungin in the CaFT, a structurally unrelated GS inhibitor, ergokonin A [67], was tested, revealing both *FKS1* and *RHO1* subunits of the GS enzyme complex at the highest concentration tested (Fig. S5A) as further confirmed by spot test (Fig. S5C).

Brefeldin A (BFA) is a reversible inhibitor of secretory vesicle trafficking. Its primary molecular targets are a class of ARF (ADP-ribosylation factor)-GEF (guanine nucleotide exchange factor) complexes. In *S. cerevisiae,* BFA binds to the contact interface between ScArf1p (an ARF) and ScSec7p (a GEF), and stabilizes their transient interaction into an abortive complex, leading to depletion of the pool of GEF-ARF [68]. Although BFA was not evaluated in the ScFT, its analysis by the CaFT revealed hypersensitivity of both *SEC7* and *ARF2* HD strains in a range of ICs (Fig. S6A). Interestingly, both Sec7p and Arf2p are members of protein families. The so-called Sec7 domain is present in another paralog of Sec7p, Gea2p, in *C. albicans*. Although all three *S. cerevisiae* paralogs (ScSec7p, ScGea1p and ScGea2p) have been implicated in the MOA of BFA [68], *GEA2* displayed no HI against BFA in the CaFT (Fig. S6A) or in the liquid assays (Fig. S6B). Similarly, ARF proteins in *C. albicans* comprise a large family. Those represented in the CaFT pool, in addition to *ARF2*, include *ARL1* and *ARL3*. *ARL1* displayed only minor dose-dependent HI against BFA (Fig. S6A), as confirmed in the liquid assays (Fig. S6B), while the *ARL3* strain showed no hypersensitivity. Despite the complexity of ARF and GEF (Sec7) protein families in *C. albicans*, our results clearly indicate that Arf2p and Sec7p are the functional partners and are most susceptible to BFA. (Note that *ARF1*, *orf19.6447*, was not represented in the CaFT strain pool.)

**CaFT Profiling of Inhibitors of Protein Complexes**

Cytochalasin D binds to the end of the actin filaments inhibiting both association and dissociation of actin subunits. It inhibits two independent *in vitro* actin nucleation mechanisms in *S. cerevisiae*: with formin (ScBni1p) and profilin (ScPfy1p) driving barbed end nucleation to form actin cables [69], and with the ARP2/3 complex facilitating branching of linear actin filaments to produce cortical actin structures [70]. The CaFT revealed that the latter mechanism is markedly perturbed by the inhibitor *in vivo*, as six of the seven subunits of the ARP2/3 complex displayed significant HI against cytochalasin A, while the *BNI1* and *PFY1* HD strains were largely unaffected (Fig. S7). Although these results may suggest preferential inhibition of this specific mode of actin polymerization *in vivo*, it is equally possible that polymerization catalyzed by a stoichiometrically perturbed ARP2/3 complex, as a result of heterozygosity of the coding genes, is more susceptible to chemical inhibition by cytochalasin D. In fact, biochemical studies demonstrated that the stability of the yeast ARP2/3 complex is compromised by deleting individual subunits [71]. There is also an indication that the TCP chaperone complex, involved in folding of the actin and the tubulin monomers, is noticeably susceptible to cytochalasin D (Fig. S7), likely due to a decrease in the level of the native actin monomer. Interestingly, *KAP120* (a karyopherin-type nuclear transport factor) displayed a highly specific and reproducible HI against cytochalasin D, despite no known functional connection to the process of actin polymerization.

Roridin A and verrucarin A are structurally related mycotoxins and potent inhibitors of translation initiation [72], a complex and sequential process involving a ternary complex of eIF2, GTP and Met-tRNA_i_^Met^ binding to the 40S ribosome and mRNA through eIF3 activity, in conjunction with eIF1, elF1A and eIF5 [73]. The MOA of roridin A and verrucarin A is clearly identified in the CaFT, as both elicited specific hypersensitivity of heterozygotes corresponding to four of the five subunits of eIF3, *RPG1*, *PRT1*, *NIP1* and *TIF34* (Fig. S8). In addition, the gene encoding eIF1A, *TIF11*, also displayed modest HI against both toxins (Fig. S8), unlike eIF1 (*SUI1*) or eIF2 (*SUI2*, *SUI3* and *EIF2*) (Table S1). Furthermore, the strain corresponding to the only subunit of eIF3 that escaped detection in the CaFT for hypersensitivity, *TIF35*, is itself severely haploinsufficient under the normal growth conditions (Fig. S8B). Although it is not clear how the stoichiometry of eIF3 is regulated in *C. albicans*, the observed HI of *TIF35* might indicate a critical role of the corresponding protein in maintaining the integrity of eIF3.

**CaFT Profiling of Analogs of Cytosine, Uracil and Adenosine**

When 5-FU, 5-FC and tubercidin were tested in the CaFT, their profiles contain multiple hypersensitive and resistant strains, and relate to each other. This relatedness was evident when the top 30 most affected strains (20 hypersensitive, 10 resistant) from each profile were compared. Due to overlapping, the combined profiles (of the most responsive strains) comprise of 48 strains, majority of which correspond to genes involved in ribosomal RNA processing and biogenesis (highlighted by black boxes, Fig. S9A). For comparison, the responses of these 48 strains to 6-AU, another analog of uracil, in the CaFT were also included. With the only exception of *FUR1*, there is no similarity between profiles of 6-AU and the three analogs described above – a clear indication that the ribosomal processing defects are specifically associated with 5-FC, 5-FU and tubercidin. Incidentally, both 5-FC and 5-FU, once incorporated into rRNA, could potentially block the base rotation required for psuedouridines (Fig. 6B), part of the posttranscriptional modifications of pre-rRNA, while 6-AU does not. It is, however, not clear how tubercidin, an analog of adenosine, exerts its effects on pre-rRNA processing and ribosomal biogenesis.

The CaFT profile of tubercidin contains an interesting resistant strain, the corresponding gene (*NNT1*) of which encodes a H^+^-dependent nucleoside transporter that is absent in *S. cerevisiae*. We postulated that the resistance of this strain is likely due to reduced uptake of tubercidin resulted from heterozygosity of the nucleoside transporter gene. In *Leishmania donovani*, mutations in the orthologous nucleoside transporter confer resistance to this compound [74]. We used a conditional shut-off strain for *NNP1* [6] to determine if it confers specific resistance when the expression is repressed. This was indeed observed under the repressing conditions (Fig. S9B, bottom panel). However, we also noted that under the non-pressing conditions, this strain is slightly more susceptible to tubercidin – enhanced uptake of this compound caused likely by overexpression of *NNT1* under the control of the TET promoter. This dimorphic response is specific to tubercidin, since this strain showed no differential susceptibility to 5-FU or 5-FC under the non-repressing and the repressing conditions (Fig. 6C and 6D).

**References**

(Note that only those appear in the supplementary results are listed here.)

1. Bolard J (1986) How do the polyene macrolide antibiotics affect the cellular membrane properties? Biochim Biophys Acta. 864:257-304.
2. Eckert V, Blank M, Mazhari-Tabrizi R, Mumberg D, Funk M, Schwarz RT. (1998) Cloning and functional expression of the human GlcNAc-1-P transferase, the enzyme for the committed step of the dolichol cycle, by heterologous complementation in *Saccharomyces cerevisiae*. Glycobiology. 8:77-85.
3. Bonilla M, Nastase KK, Cunningham KW (2002) Essential role of calcineurin in response to endoplasmic reticulum stress. EMBO J 21:2343-2353.
4. Nagiec MM, Nagiec EE, Baltisberger JA, Wells GB, Lester RL, Dickson RC (1997) Sphingolipid synthesis as a target for antifungal drugs. Complementation of the inositol phosphorylceramide synthase defect in a mutant strain of *Saccharomyces cerevisiae* by the AUR1 gene. J Biol Chem 272:9809-9817.
5. Kartsonis NA, Nielsen J, Douglas CM (2003) Caspofungin: the first in a new class of antifungal agents. Drug Resist Updat 6:197-218.
6. Douglas CM, D'Ippolito JA, Shei GJ, Meinz M, Onishi J, Marrinan JA, Li W, Abruzzo GK, Flattery A, Bartizal K, Mitchell A, Kurtz MB (1997) Identification of the FKS1 gene of *Candida albicans* as the essential target of 1,3-beta-D-glucan synthase inhibitors. Antimicrob Agents Chemother 41:2471-2479.
7. Kondoh O, Tachibana Y, Ohya Y, Arisawa M, Watanabe T. (1997) Cloning of the RHO1 gene from *Candida albicans* and its regulation of beta-1,3-glucan synthesis. J Bacteriol. 179:7734-7741.
8. Onishi J, Meinz M, Thompson J, Curotto J, Dreikorn S, Rosenbach M, Douglas C, Abruzzo G, Flattery A, Kong L, Cabello A, Vicente F, Pelaez F, Diez MT, Martin I, Bills G, Giacobbe R, Dombrowski A, Schwartz R, Morris S, Harris G, Tsipouras A, Wilson K, Kurtz MB. (2000) Discovery of novel antifungal (1,3)-beta-D-glucan synthase inhibitors. Antimicrob. Agents Chemother. 44:368-377.
9. Peyroche A, Antonny B, Robineau S, Acker J, Cherfils J, Jackson CL. (1999) Brefeldin A acts to stabilize an abortive ARF-GDP-Sec7 domain protein complex: involvement of specific residues of the Sec7 domain. Mol Cell. 3:275-285.
10. Sagot I, Rodal A, Moseley J, Goode B, Pellman D (2002) An actin nucleation mechanism mediated by Bni1 and Profilin. Nature Cell 4:626-631.
11. Winter D, Lechler T, Li R (1999) Activation of the yeast Arp2/3 complex by Bee1p, a WASP-family protein. Current Biology 9:501-504.
12. Winter D. Choe EY, Rong L (1999) Genetic dissection of the budding yeast Aep2/3 complex: A comparison of the *in vivo* and structural roles of individual subunits. Proc Natl Acad Sci USA 96:7288-7293.
13. Cundliffe, E and Davies, JE (1977) Inhibition of initiation, elongation, and termination of eukaryotic protein synthesis by trichothecene fungal toxins. Antimicrob Agents Chemother 11:491–499.
14. Valasek L, Nielsen KH, Hinnebusch AG. (2002) Direct eIF2-eIF3 contact in the multifactor complex is important for translation initiation *in vivo*. EMBO J. 21:5886-5898.
15. Vasudevan G, Ullman B, Landfear SM (2001) Point mutations in a nucleoside transporter gene from *Leishmania donovani* confer drug resistance and alter substrate selectivity. Proc Natl Acad Sci U S A. 98(11):6092-6097.

**Figure Legends**

**Fig S1**. CaFT profiles of imidazoles (ketoconazole, clotrimazole, econazole and sulconazole) and a triazole (voriconazole).

**Fig. S2**. CaFT profiles of inhibitors of enzymes involved in ergosterol biosynthesis. *A*. Spot tests of terbinafine, lovastatin and dyclonine against heterozygous deletion strains corresponding to enzymes involved in ergosterol biosynthesis and the control (*HIS3*). CaFT profiles of terbinafine (*B*), lovastatin (*C*) and dyclonine (*D*).

**Fig. S3**. CaFT profiles of amphotericin B (A), and the two ergosterol analogs, ECC69 and ECC1384 (B). *C*. To compare the profiles of these three compounds, the top 30 most sensitive strains to each compound were selected and the three sets were combined for comparison. Highlighted in red are z-scores greater than 3.000. Strains whose corresponding genes are involved in the membrane function and metabolism are highlighted.

**Fig. S4**. CaFT profiles of tunicamycin and aureobasidin A. *A*. CaFT profiles of tunicamycin at multiple ICs. *B*. Top 10 most hypersensitive strains identified in the CaFT with tunicamycin at the concentrations indicated, with z-scores greater than 4.000 highlighted in bold. *C*. Spot tests with selected strains against tunicamycin. Both the *HIS3* and *SEC7* (*orf19.5947*) strains are controls. *D*. CaFT profiles of aureobasidin A, with highlighted strains: *AUR1* = *orf19.1945*, *orf19.839*, *ORM1* = *orf19.5751*, *KRE8* = *orf19.942*, and *RVB2* = *orf19.6539*. *E*. Spot tests with heterozygous deletion strains for genes involved in sphingolipid biosynthesis against aureobasidin A. Note that the *LCB2* (*orf19.5027*) strain (the asterisk) was hyper-variable in the CaFT; i.e., it responded to different inhibitory compounds with no chemical structural relatedness, hence it normalized z-score was minimal (see text for detail). Note, the *IPT1* (*orf19.4769*) strain (the underlined) was not in the CaFT strain pool.

**Fig. S5**. CaFT profiles of the β-1,3-glucan synthase inhibitors, caspofungin and ergokonin A (*A*). *B*. Spot tests of caspofungin and voriconazole. The results of one- and two-day growth are shown for comparison between hypersensitivity of the *FKS1* and *RHO1* heterozygous deletion strains against caspofungin and that of the *ERG11*, *NCP1* and *CDR1* strains against voriconazole. While the latter hypersensitivity persisted during the 2-day incubation, the former was readily seen only after 1-day incubation. *C*. Spot tests of caspofungin (at higher concentrations) and ergokonin A reproduce hypersensitivity of the *FKS1* and *RHO1* strains observed in the CaFT.

**Fig. S6**. CaFT profiling and characterization of brefeldin A (BFA). *A*. CaFT profiles of BFA, with relevant strains indicated. The colored symbols indicate strains with significant hypersensitivity to BFA: *SEC7* (*orf19.5947*), *ARF2* (*orf19.5964*), *ARL1* (*orf19.4603*), and *CDR1* (*orf19.6000*, an efflux pump for BFA, as shown by Sanglard et al [21]). Other highlighted stains include: *GEA2* (*orf19.1712*) and *ARL3* (*orf19.2297)*. *B*. Independent confirmation of hypersensitivity of the *SEC7* and *ARF2* strains against BFA in liquid assays. Since BFA displays poor activity on solid medium, liquid assays were employed to determine hypersensitivity. The liquid (RPMI) cultures (in a 96-well plate) of the selected strains were first incubated in the absence or presence of BFA at the concentrations indicated for 6 hours (reaching exponential growth) and then diluted to an OD_600_ of 0.005. The fresh cultures with or without BFA were incubated for another 14 hours, after which growth was measured at 2 hour intervals for 4 hours.

**Fig. S7**. CaFT profiles of cytochalasin D. Highlighted are strains corresponding to 1) subunits of the ARP2/3 complex [*ARP2* (*orf19.7292*), *ARP3* (*orf19.2289*), *ARC40* (*orf19.3873*), *ARC35* (*orf19.2437*), *ARC19* (*orf19.3251*), *ARC18* (*orf19.121*) and *ARC15* (*orf19.6151*). Note that *ACT1* (*orf19.5007)* heterozygous deletion strain was not included in the CaFT pool.], 2) two genes involved in formation of actin cables, *BNI1* (*orf19.4927*) and *PFY1* (*orf19.5076*), and 3) additional hypersensitive strains, *CCT3* (*orf19.4004*), *CCT6* (*orf19.3126*), *CTT8* (*orf19.6099*), *CMD1* (*orf19.4413*), *KAP120* (*orf19.7177*) and *CDR1* (*orf19.6000*, the encoded pump is likely involved in efflux of this compound).

**Fig. S8**. CaFT profiles of mycotoxins. *A.* CaFT profiles of roridin A and verrucarin A with highlighted strains for subunits of eIF3 [*RPG1* (*orf91.6345*), *PRT1* (*orf19.6584*), *NIP1* (*orf19.4635*), *TIF34* (*orf19.2967*) and *TIF35* (*orf19.7236*)] and eIF1A [*TIF11* (*orf19.5351*)]. *B*. Spot tests of heterozygous deletion strains corresponding to the five subunits of eIF3 and eIF1A, together with strains for *HIS3* and the two subunits of eIF2: *SUI2* (*orf19.6213*) and *SUI3* (*orf19.7161*). Note that *TIF35* is severely haploinsufficient under normal growth conditions.

**Fig. S9**. CaFT profiles of 5-FU, 5-FC, tubercidin and 6-AU, and characterization of *C. albicans* nucleoside transporter Nnt1p. *A*. CaFT profiling of 5-FU, 5-FC, tubercidin and 6-AU. The selection of strains was based on their responses to 5-FU, 5-FC and tubercidin (see text for detail) with orf19 designations and *S. cerevisiae* orthologous genes indicated. The z-scores are shown with those greater than 3.000 highlighted in red, and lower than -3.000 in blue (black filling indicating ND = not determined). Black boxes indicate strains representing genes involved in pre-rRNA processing and ribosomal biogenesis. The experimental conditions are: 5-FC: 1, 0.006 µg/ml (F=0.89); 2, 0.010 µg/ml (F=0.61); 3, 0.014 µg/ml (F=0.57), 5-FU: 4, 2.5 µg/ml (F=0.94); 5, 4.5 µg/ml (F=0.75); 6, 3.5 µg/ml (F=0.82); tubercidin: 7, 0.80 µg/ml (F=0.89); 8, 0.95 µg/ml (F=0.80); 9, 1.15 µg/ml (F=0.69); and 6-AU: 10, 0.70 µg/ml (F=0.88); 11, 0.90 µg/ml (F=0.77); 12, 1.05 µg/ml (F=0.72). The green box highlights three resistant strains, the corresponding genes of which are involved in the uptake or metabolism of 5-FC/5-FU or tubercidin. *B*. Characterization of selected conditional shut-off strains under the non-repressing (top) and the repressing (bottom) conditions against tubercidin. Note that only the *NNT1* strain displays dimorphic responses to tubercidin; i.e., increased susceptibility under the non-repressing conditions (likely due to overexpression of the transporter; i.e., enhanced uptake of the compound) and resistance under the repressing conditions (i.e., reduced uptake when the transporter is depleted).

**Fig. S10**. Dose-dependent inhibition of *in vitro* polymerization of bovine tubulin by nocodazole (*A*), ECC85 (*B*), ECC248 (*C*) and ECC275 (*D*). Polymerization was monitored at 1 minute intervals for 60 minutes.
